# Surgical strategies for spontaneous intracerebral hemorrhage: a Bayesian network meta-analysis of randomized controlled trials

**DOI:** 10.3389/fneur.2026.1833237

**Published:** 2026-06-16

**Authors:** Yifan Zhou, Yanhua Wei, Cheng Yu, Ruilin Li, Chengyang Su

**Affiliations:** 1 Department of Neurosurgery, The Second People's Hospital of Baiyin City, Baiyin, Gansu, China; 2 Department of Anesthesiology, Lanzhou University Second Hospital, Lanzhou, Gansu, China; 3 Department of Neurology, The First People's Hospital of Lanzhou City, Lanzhou, Gansu, China

**Keywords:** Bayesian network meta-analysis, conservative medical treatment, craniotomy, decompressive Craniectomy, endoscopic surgery, intracerebral hemorrhage, minimally invasive puncture surgery

## Abstract

**Study Design:**

A Systematic Review and Bayesian Network Meta-Analysis.

**Objective:**

To compare the efficacy and perioperative outcomes of conservative medical treatment (CMT) and surgical interventions—including decompressive craniectomy (DC), craniotomy (CC), endoscopic surgery (ES), and minimally invasive puncture surgery (MIPS)—in patients with spontaneous intracerebral hemorrhage (ICH).

**Background:**

Spontaneous intracerebral hemorrhage (ICH) is a severe neurological emergency associated with substantial mortality and long-term disability. Although several surgical strategies have been developed to reduce hematoma burden and secondary brain injury, the comparative effectiveness and perioperative trade-offs among different interventions remain controversial. Direct head-to-head randomized evidence comparing multiple surgical strategies is limited, complicating evidence-based clinical decision-making.

**Methods:**

We conducted a systematic review and network meta-analysis (NMA) in accordance with PRISMA 2020 and PRISMA-NMA guidelines. PubMed, Web of Science, and Cochrane Library were searched from inception to January 2026 for randomized controlled trials comparing CMT, DC, CC, ES, and MIPS in patients with spontaneous ICH. Primary outcomes included 6-month mortality and good functional outcome at 6 months. Secondary outcomes included hematoma clearance rate, operative time, intraoperative blood loss, and length of hospital stay. Pairwise meta-analysis was performed using Stata 18.0, and Bayesian NMA was conducted in R 4.3.1 using the gemtc and BUGSnet packages. Surface under the cumulative ranking curve (SUCRA) values were used to rank interventions.

**Results:**

Eighteen randomized controlled trials involving 4,497 patients were included. For good functional outcome at 6 months, MIPS (SUCRA = 87.0) and ES (SUCRA = 84.6) ranked highest and were significantly superior to CC and CMT, whereas no significant difference was observed between MIPS and ES. For 6-month mortality, DC probabilistically ranked highest (SUCRA = 81.5), although most pairwise comparisons did not reach statistical significance. Regarding perioperative outcomes, both ES and MIPS significantly reduced operative time and intraoperative blood loss compared with CC, with MIPS showing the largest reductions. ES achieved higher hematoma clearance rates and shorter hospital stay, whereas MIPS demonstrated lower hematoma clearance.

**Conclusion:**

MIPS and ES may provide advantages in functional recovery and perioperative burden in spontaneous ICH, whereas DC may offer potential survival benefit, although current evidence remains uncertain. Clinical decision-making should balance long-term outcomes against perioperative trade-offs and be individualized according to disease severity and patient-specific risk factors. Additional high-quality multicenter randomized trials are needed to clarify the role of DC and define optimal indications for each surgical strategy.

## 1 Introduction

Spontaneous intracerebral hemorrhage (ICH) is a devastating subtype of stroke caused by non-traumatic bleeding into the brain parenchyma and accounts for approximately 10–20% of all strokes worldwide (1, 2). Despite advances in neurocritical care and surgical techniques, spontaneous ICH remains associated with high mortality and severe long-term neurological disability. Reported mortality rates approach 30–40% within the 1 month, and many survivors experience substantial neurological impairment and reduced quality of life. Advanced age, hypertension, cerebral amyloid angiopathy, anticoagulant use, and vascular abnormalities are important risk factors for spontaneous ICH and are associated with worse clinical outcomes in selected populations. Hematoma expansion, mass effect, elevated intracranial pressure, and secondary brain injury are major contributors to poor outcomes.

Management strategies for spontaneous ICH remain controversial. Conservative medical treatment (CMT) is commonly applied in patients with relatively small hematoma volume, mild neurological deficits, or high operative risk. In contrast, surgical intervention is generally considered in patients with neurological deterioration, significant mass effect, or large hematoma burden. Common surgical approaches include decompressive craniectomy (DC), conventional craniotomy (CC), endoscopic surgery (ES), and minimally invasive puncture surgery (MIPS) (3–5).

Each surgical strategy has potential advantages and limitations. DC may rapidly reduce intracranial pressure and relieve mass effect but is highly invasive and associated with considerable perioperative burden. CC allows direct hematoma evacuation and hemostasis but may increase tissue injury because of extensive surgical exposure (6). Minimally invasive approaches such as ES and MIPS may reduce operative trauma, operative time, and intraoperative blood loss; however, concerns remain regarding adequacy of hematoma evacuation and long-term functional benefit (7).

Although numerous randomized controlled trials (RCTs) have investigated surgical management for spontaneous ICH, most studies have focused on individual procedures or limited pairwise comparisons. Consequently, the relative comparative effectiveness among multiple competing surgical strategies remains unclear. Traditional pairwise meta-analysis cannot simultaneously compare all available interventions when direct evidence is incomplete.

Network meta-analysis (NMA) enables integration of both direct and indirect evidence across multiple interventions within a unified analytical framework and allows probabilistic ranking of treatment strategies. Therefore, we conducted a systematic review and Bayesian NMA restricted to randomized controlled trials to compare conservative management and surgical strategies (CMT, DC, CC, ES, and MIPS) for spontaneous intracerebral hemorrhage. Our aim was to evaluate comparative effectiveness, perioperative trade-offs, and current evidence gaps to support individualized clinical decision-making and future guideline development.

## 2 Methods

### 2.1 Study design, reporting, and registration

This study is a systematic review and network meta-analysis (NMA) designed to compare the effectiveness and safety of different surgical interventions and conservative medical treatment for spontaneous ICH. The review was conducted and reported in accordance with the PRISMA statement and its extension for network meta-analysis (PRISMA-NMA), and methodological guidance from the Cochrane Handbook was followed to enhance transparency and reproducibility (8). The study protocol was prospectively registered in PROSPERO (CRD420261338464), and the research question, eligibility criteria, outcomes, and statistical plan were pre-specified prior to study initiation.

### 2.2 Information sources and search strategy

We systematically searched PubMed, Web of Science, and the Cochrane Library from inception to January 2026. The search strategy combined three components: (1) condition-related terms (e.g., “intracerebral hemorrhage,” “non-traumatic,” and synonyms); (2) intervention-related terms (e.g., “conservative medical treatment,” “decompressive craniectomy,” “craniotomy,” “endoscopic surgery,” “minimally invasive puncture surgery,” and abbreviations CMT, DC, CC, ES, and MIPS); and (3) study design terms (e.g., “randomized controlled trial,” “randomized,” “clinical trial”). Both controlled vocabulary and free-text terms were used. The full search strategy is provided in the Supplement. To minimize missed studies, reference lists of relevant systematic reviews and pairwise meta-analyses were screened, and potentially eligible studies were hand-checked.

### 2.3 Study selection

Two reviewers (YZ, CY) independently screened records in two stages (title/abstract screening followed by full-text review) according to pre-defined eligibility criteria. For duplicate publications or multiple reports from the same trial/cohort, the most complete report (with more comprehensive follow-up or outcomes) was prioritized; if different reports contributed different outcomes, data were integrated without double-counting participants. Disagreements were resolved through discussion with a third reviewer (CYS).

### 2.4 Eligibility criteria

We included only randomized controlled trials (RCTs) and excluded case reports, reviews, conference abstracts, letters, opinion pieces, protocols, and other non-original research.

#### 2.4.1 Population

Patients with imaging-confirmed spontaneous intracerebral hemorrhage (ICH), particularly supratentorial ICH. Studies involving traumatic intracerebral hemorrhage, traumatic brain injury, isolated subdural hematoma, epidural hematoma, subarachnoid hemorrhage, or hemorrhagic transformation of ischemic stroke were excluded.

#### 2.4.2 Interventions/comparators

Conservative medical treatment (CMT), decompressive craniectomy (DC), craniotomy (CC), endoscopic surgery (ES), and minimally invasive puncture surgery (MIPS). Comparators could be CMT or another surgical strategy. Combination interventions were excluded if the independent effect of the target procedure could not be separated.

#### 2.4.3 Follow-up

Eligible studies were required to report or allow extraction of 6-month follow-up data for the primary outcomes.

#### 2.4.4 Outcomes

The primary outcomes were (1) 6-month mortality and (2) good functional outcome at 6 months. Good functional outcome was extracted as defined in each study (e.g., mRS ≤ 2, GOS 4–5, or study-specified thresholds); if multiple thresholds were reported, the pre-specified primary threshold was used (9, 10).

Secondary outcomes included hematoma clearance rate (reported as % or percentage points), operative time, intraoperative blood loss, and length of hospital stay. Continuous outcomes were synthesized using original units when possible (minutes, milliliters, days, and percentage points). If units were not comparable and could not be converted, standardized mean difference (SMD) was considered, and robustness was assessed in sensitivity analyses.

### 2.5 Data extraction and handling of missing data

Two reviewers independently extracted data and cross-checked results. Extracted information included first author, publication year, country/region, study design, sample size, follow-up duration, intervention and comparator details, and outcome data. When multiple follow-up time points were reported, the pre-specified primary time point was prioritized; otherwise, the final follow-up time point was used.

For missing or incomplete variance data (e.g., SD/SE not reported), we followed Cochrane Handbook guidance. When confidence intervals, *P* values, or t statistics were available, SD/SE was derived using standard formulas. When only medians and interquartile ranges/ranges were reported, established conversion methods were used when appropriate (11, 12). If reliable estimation was not possible, study authors were contacted; otherwise, the study was excluded from quantitative synthesis for that outcome and summarized qualitatively. All rules were pre-specified, and sensitivity analyses were conducted when needed.

### 2.6 Risk of bias assessment

Risk of bias in included RCTs was assessed using a Cochrane-recommended tool, covering the randomization process, deviations from intended interventions, outcome measurement, missing outcome data, selective reporting, and other sources of bias. Each domain was judged as low risk, high risk, or some concerns/unclear risk. Two reviewers assessed risk of bias independently, with disagreements adjudicated by a third reviewer. Risk-of-bias plots were generated using RevMan 5.4 or equivalent tools (13, 14).

### 2.7 Statistical analysis, inconsistency assessment, and presentation

Before conducting the NMA, we assessed the transitivity assumption by systematically comparing potential effect modifiers across treatment comparisons, including baseline neurological severity, hematoma volume, hematoma location, timing of intervention, age, and surgical technique. These characteristics were extracted from each included trial and summarized by treatment node in Supplementary Table 2. We considered transitivity acceptable if the distributions of these key clinical and methodological factors were broadly comparable across comparisons and if no treatment node was exclusively represented by a clearly distinct patient subgroup. For outcomes with direct comparisons, we first performed pairwise meta-analyses in Stata 18.0. Continuous outcomes were summarized as mean differences (MD) (or SMD when required), and dichotomous outcomes as odds ratios (OR) (or risk ratios when appropriate), each with 95% confidence intervals (95% CI). Statistical heterogeneity was quantified using I^2^; random-effects models were used when heterogeneity was substantial, and potential sources were explored when feasible (15).

NMA was conducted in R 4.3.1 using the gemtc and BUGSnet packages under a consistency framework, combining direct and indirect evidence to estimate relative effects between any two interventions (16). Prior to NMA, the transitivity assumption was assessed clinically and methodologically, focusing on baseline severity, imaging characteristics, timing of intervention, comparator settings, and outcome measurement. Statistical inconsistency was evaluated using a global inconsistency test and local approaches (e.g., node-splitting). Where inconsistency was suspected, network structure and clinical differences were examined, and sensitivity analyses were performed when necessary (17).

Treatment ranking was summarized using SUCRA values and ranking plots. Because outcome reporting differed across trials, separate networks were constructed for each outcome. For primary outcomes, the network included CMT, DC, CC, ES, and MIPS. For surgery-related secondary outcomes (hematoma clearance rate, operative time, intraoperative blood loss, and length of hospital stay), DC lacked extractable data in the included RCTs; thus, the corresponding networks included only interventions with available data (typically CC, ES, and MIPS). When networks contained few interventions (e.g., three-node networks), relative effects (forest plots and summary comparison plots) were emphasized to avoid over-interpretation of rankings.

### 2.8 Sensitivity analyses

Sensitivity analyses were performed to evaluate the robustness of the main findings. First, all included studies were re-evaluated after correction of the disease classification to confirm that the study populations involved spontaneous intracerebral hemorrhage. Second, primary outcomes were reanalyzed after excluding studies judged to be at high risk of bias. Third, functional outcome definitions and major clinical effect modifiers, including hematoma location, baseline severity, hematoma volume, and timing of intervention, were qualitatively assessed. Formal subgroup network meta-analysis or meta-regression was not performed when data were sparse or incompletely reported.

### 2.9 Certainty of evidence assessment

The certainty of evidence for the primary and secondary outcomes was qualitatively assessed based on GRADE principles. The assessment considered major domains including risk of bias, inconsistency/transitivity, indirectness, imprecision, and potential publication bias across the network estimates. Given the complexity and sparsity of several treatment networks, the certainty assessment was performed qualitatively rather than through a fully formalized CINeMA framework. Overall certainty ratings were categorized as high, moderate, low, or very low.

## 3 Results

### 3.1 Study selection and characteristics

**Figure 1:**
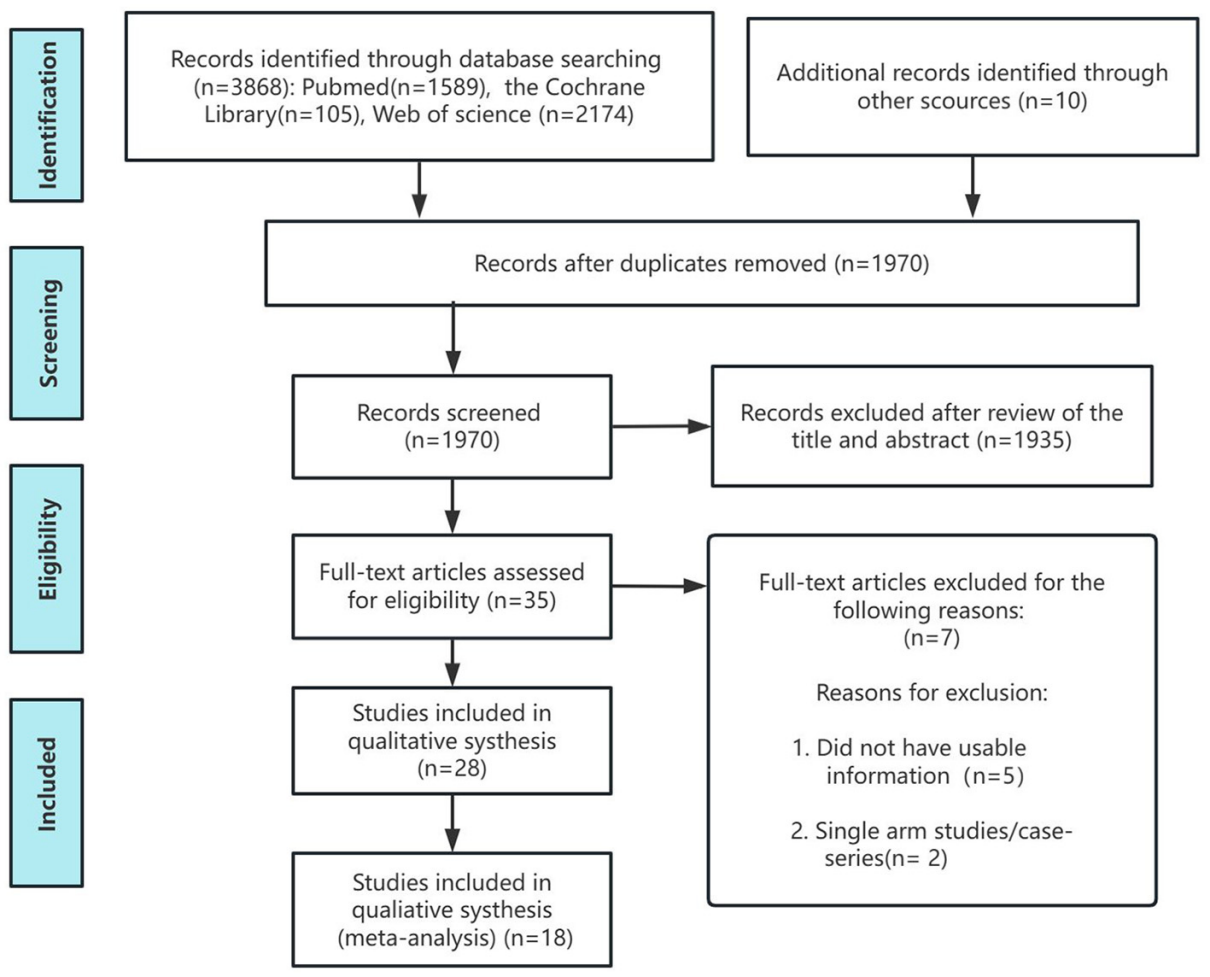
Flow chart of the selection process for relative studies in meta-analysis.

The study selection process is shown in Figure 1. The initial search yielded 3,868 records; after removing duplicates, 1,970 records were screened by title and abstract. Full texts of potentially relevant studies were assessed and excluded based on pre-specified criteria. For example, the study by L. B. Morgenstern et al. (18) was excluded due to incomplete key data. The trial by Daniel F. Hanley et al. (19) was excluded because the primary outcomes and follow-up duration were 12 months rather than the pre-specified 6 months (19). In addition, studies by Chengjia Gui (20) and Lei Luan (21) were excluded because follow-up was limited to 3 months. Ultimately, 18 RCTs involving 4,497 participants were included in the quantitative synthesis (2, 7, 22–37). The interventions compared included CMT, DC, CC, ES, and MIPS.

**Figure 2:**
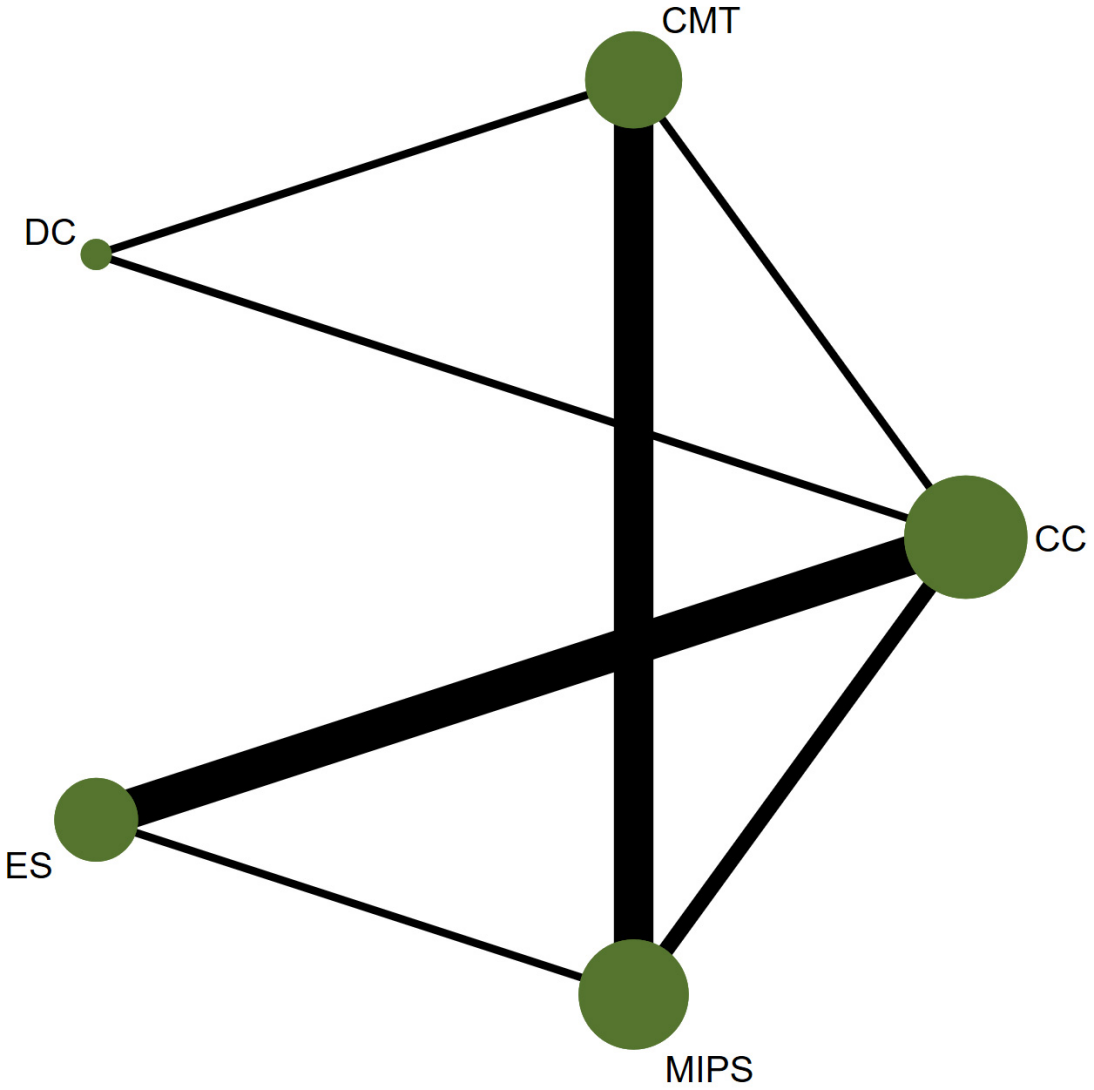
The network plot of all trials (CMT, conservative medical treatment; DC, decompressive craniectomy; CC, craniotomy; ES, endoscopic surgery; MIPS, minimally invasive puncture surgery).

The network geometry is presented in Figure 2. Supplementary Table 1 compares deviance information criterion (DIC) values between the consistency and inconsistency models; DIC comparisons and node-splitting analyses did not identify statistically significant inconsistency within the network. However, we acknowledge that these statistical approaches cannot fully account for underlying clinical and methodological heterogeneity across studies. Therefore, the plausibility of the network was additionally evaluated through qualitative assessment of key clinical effect modifiers, including hematoma location, baseline severity, hematoma volume, timing of intervention, and surgical characteristics (Supplementary Table 2). Node-splitting analyses did not detect significant inconsistency (*P* > 0.05), indicating that estimates were broadly coherent under both models. Supplementary Figures 1 and 2 show the forest plots and corresponding network graphs for the primary outcomes, where node size reflects sample size and edge thickness reflects the number of direct comparisons. Supplementary Figure 3 presents funnel plots for all outcomes; visual inspection did not suggest substantial publication bias.

Baseline characteristics of included studies are summarized in Table 1. Studies were published between 2003 and 2024, with sample sizes ranging from 30 to 1,033 participants, and all had follow-up of at least 6 months. After excluding multicenter trials, Japan, South Korea, and China contributed the largest number of included studies (two from each country). Only one trial compared three surgical approaches; the remaining studies compared two strategies.

**Table 1:** The main features of the articles.

Reference	Sample size	Region	Center	Intervention	Age (mean)	Male, *n* (%)	ICH volume, mean (ml)	Time to surgery (H)	Follow up (M)
Teernstra et al. (37)	70	Netherlands	Multi	MIPS/CMT	68	40 (57)	59	< 72	6
Hattori et al. (36)	242	Japan	Multi	MIPS/CMT	61	148 (61)	44	< 24	12
Mendelow et al. (2)	1,033	Multinational	Multi	Surgery (75% CC)/CMT	62	591 (57)	38	< 72	6
Pantazis et al. (35)	108	Greece	Single	CC/CMT	61	60 (56)	56	< 8	12
Kim et al. (34)	387	Korea	Single	MIPS/CMT	66	289 (75)	23	< 168	6
Zhou et al. (33)	168	China	Multi	CC/MIPS	58	109 (65)	NA	NA	12
Mendelow et al. (32)	601	Multinational	Multi	Surgery (98% CC)/CMT	64	340 (57)	41	< 72	6
Zhang et al. (31)	51	China	Single	CC/ES	61	38 (75)	60	< 24	6
Hanley et al. (29)	96	Multinational	Multi	MIPS/CMT	61	63 (66)	46	< 72	12
Feng et al. (30)	184	China	Single	CC/ES	68	114 (62)	NA	NA	6
Bhaskar et al. (28)	61	India	Single	CC/CMT	55	37 (61)	65	< 72	6
Rasras et al. (27)	30	Iran	Single	DC/CC	59	43 (13)	47	NA	6
Deng et al. (26)	78	China	Multi	MIPS/CMT	62	48 (62)	35	NA	6
Noiphithak et al. (24)	188	Thailand	Single	CC/ES	51	130 (69)	50	< 12	6
Lv et al. (25)	128	China	Single	CC/ES	56	85 (66)	30	< 24	6
Pradilla et al. (7)	150	Multinational	Multi	MIPS/CMT	63	150 (50)	55	< 24	6
Beck et al. (23)	201	Multinational	Multi	DC/CMT	61	134 (68)	57	< 72	12
Xu et al. (22)	721	China	Multi	CC/MIPS/ES	57	497 (69)	49	< 36	6

**Figure 3:**
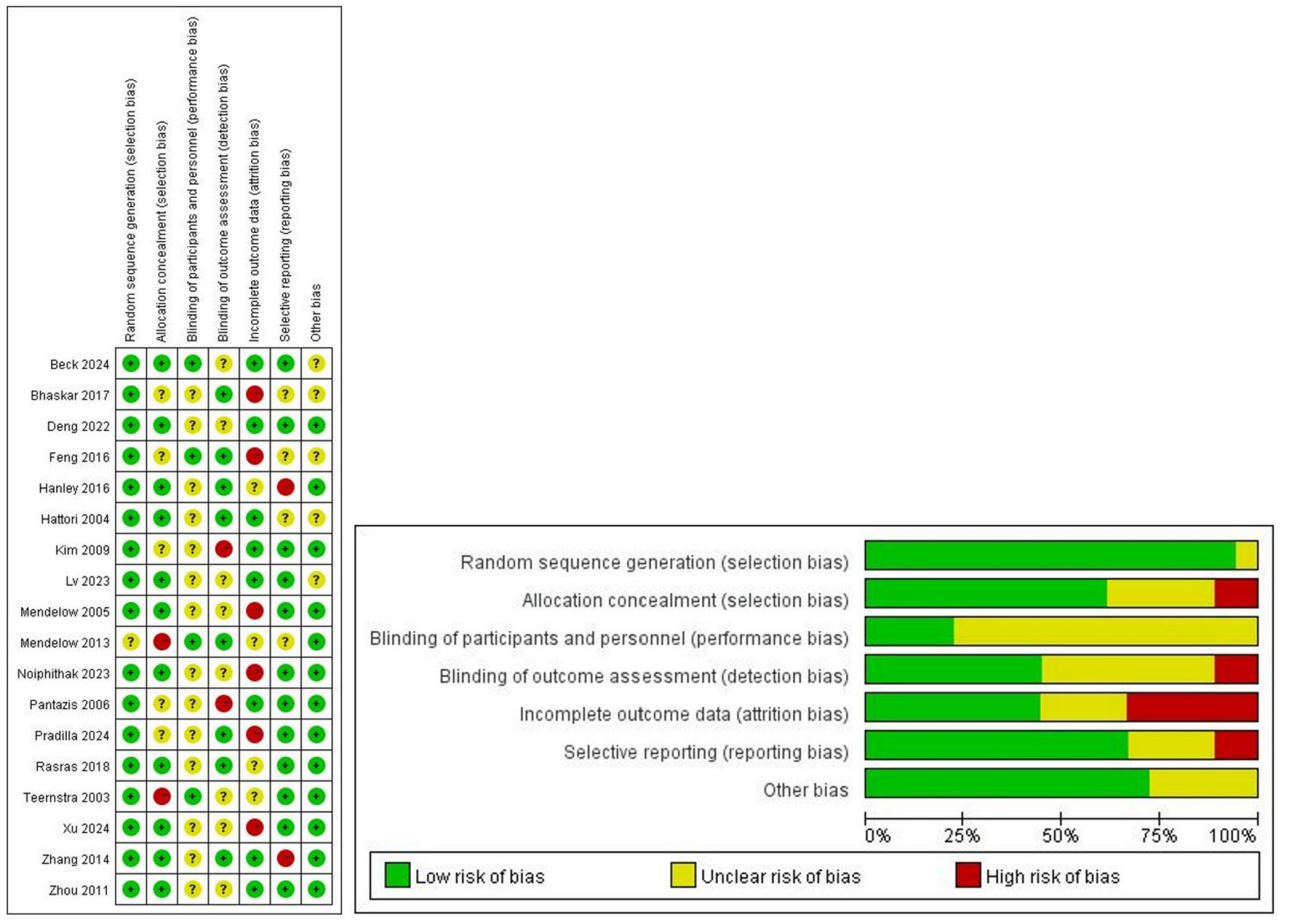
The network plot of all trials and risk of bias summary for RCTs: Reviewers' judgments about each risk of bias item per included study.

Risk of bias assessments are presented in Figure 3. Overall, reporting of sequence generation and allocation concealment was insufficient in some studies, particularly regarding the implementation of randomization and concealment procedures. In addition, several studies did not clearly report whether all pre-specified outcomes were fully reported, introducing uncertainty regarding selective reporting. Due to limited methodological details, a proportion of studies were judged as unclear risk/some concerns for selection and reporting bias. Risk of bias varied across studies, and the certainty of evidence should be considered when interpreting the findings.

### 3.2 Primary outcomes

#### 3.2.1 Good functional outcome at 6 months

**Figure 4:**
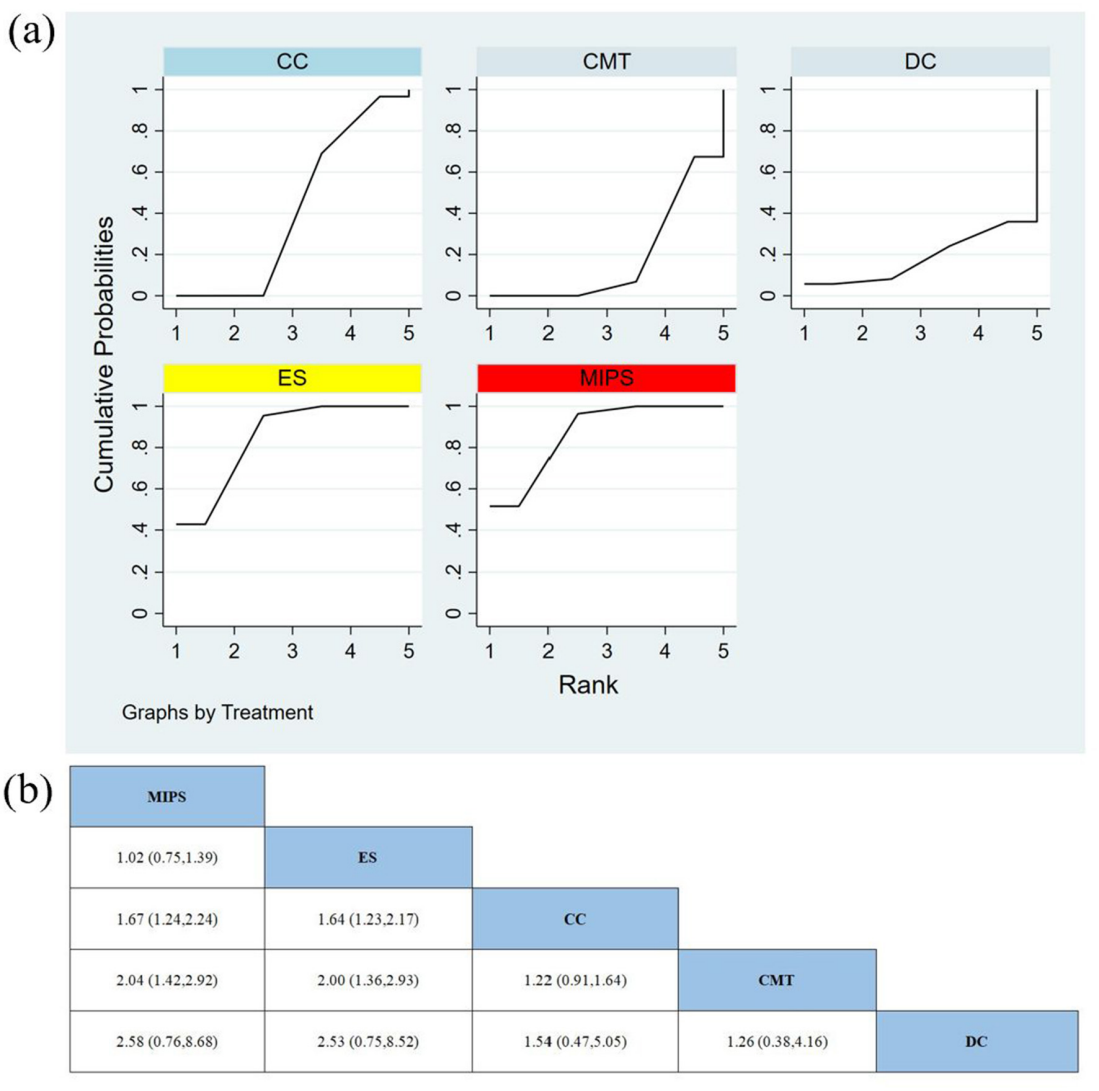
**(a)** Ranking the probability of Good functional outcome at 6 monthse. **(b)** The results of League table for Good functional outcome at 6 months.

For good functional outcome at 6 months, SUCRA rankings indicated clear differences across interventions. MIPS had the highest probability of being ranked best (Rank 1), followed by ES (Rank 2) and CC (Rank 3), whereas CMT and DC were ranked lower (Figure 4a). The corresponding SUCRA values were 87.0 for MIPS, 84.6 for ES, 41.4 for CC, 18.6 for CMT, and 18.5 for DC.

The league table results were broadly consistent with the SUCRA hierarchy (Figure 4b). Compared with CC, both MIPS (OR = 1.67, 95% CI 1.24–2.24) and ES (OR = 1.64, 95% CI 1.23–2.17) significantly increased the likelihood of achieving a good functional outcome at 6 months. Similarly, compared with CMT, MIPS (OR = 2.04, 95% CI 1.42–2.92) and ES (OR = 2.00, 95% CI 1.36–2.93) showed significant advantages. There was no statistically significant difference between MIPS and ES (OR = 1.02, 95% CI 0.75–1.39). In addition, CC did not differ significantly from CMT (OR = 1.22, 95% CI 0.91–1.64). Comparisons involving DC were imprecise with wide confidence intervals (e.g., MIPS vs DC: OR = 2.58, 95% CI 0.76–8.68; ES vs DC: OR = 2.53, 95% CI 0.75–8.52), indicating substantial uncertainty and insufficient evidence to draw definitive conclusions regarding DC for this outcome.

#### 3.2.2 6-month mortality

**Figure 5:**
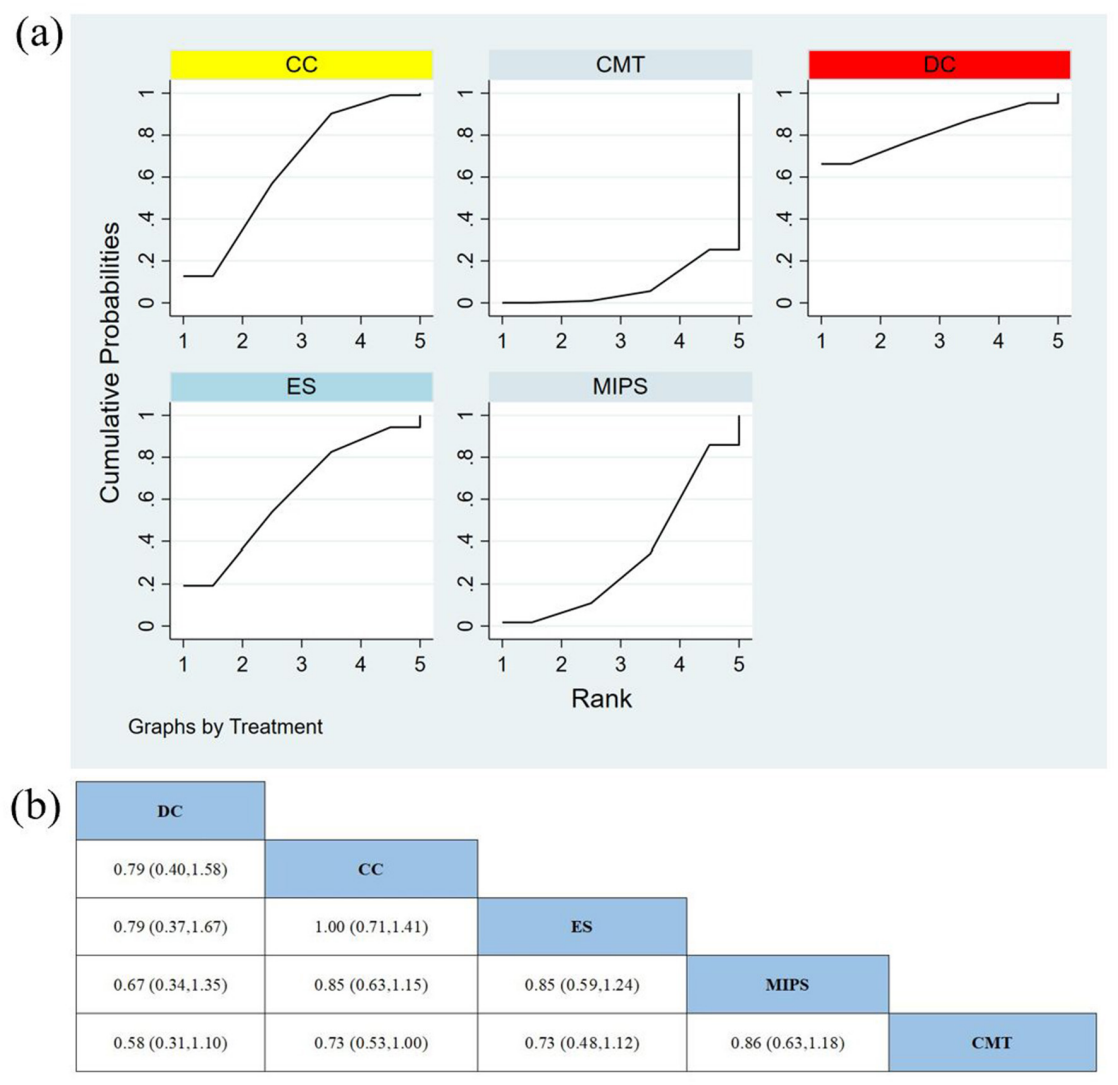
**(a)** Ranking the probability of 6-month mortality. **(b)** The results of League table for 6-month mortality.

For 6-month mortality, SUCRA rankings suggested differences in the relative hierarchy of interventions. DC had the highest probability of being ranked best (Rank 1), followed by CC (Rank 2) and ES (Rank 3), whereas MIPS and CMT were ranked lower (Figure 5a). The SUCRA values were 81.5 for DC, 64.8 for CC, 62.5 for ES, 33.2 for MIPS, and 8.0 for CMT. However, these SUCRA rankings reflect probabilistic treatment hierarchy rather than definitive clinical superiority, particularly given that most pairwise comparisons were not statistically significant.

However, the league table indicated that most pairwise comparisons did not reach statistical significance (Figure 5b). Compared with CMT, DC showed a trend toward lower mortality (OR = 0.58, 95% CI 0.31–1.10), but the difference was not statistically significant. Similarly, compared with CC, DC also showed a non-significant trend toward lower mortality (OR = 0.79, 95% CI 0.40–1.58). Notably, the comparison between CC and CMT approached statistical significance (OR = 0.73, 95% CI 0.53–1.00), suggesting a possible mortality benefit of CC vs. CMT, although this finding warrants cautious interpretation. Other comparisons were also non-significant (e.g., CC vs ES: OR = 1.00, 95% CI 0.71–1.41; MIPS vs CMT: OR = 0.86, 95% CI 0.63–1.18). Overall, while the direction of effects was broadly in line with the SUCRA ordering, uncertainty remained substantial given that many confidence intervals crossed 1.0.

### 3.3 Secondary outcomes

#### 3.3.1 Operative time

**Figure 6:**
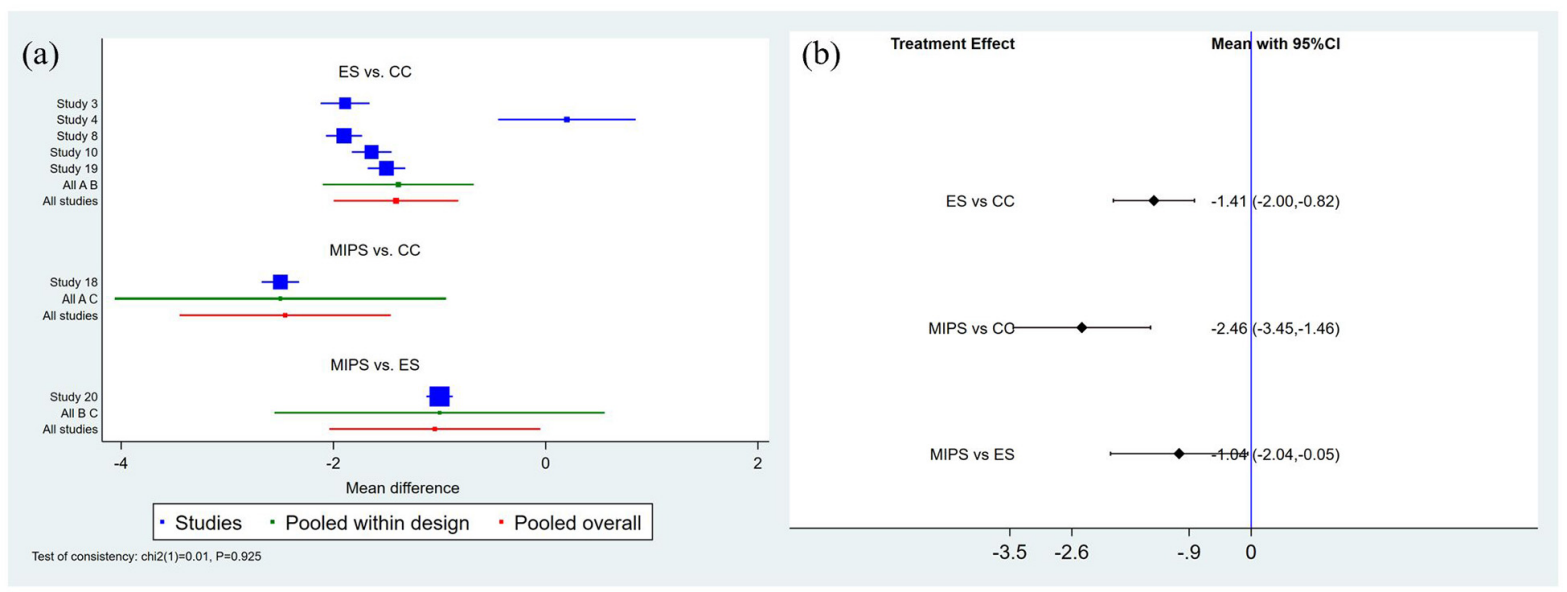
**(a)** The forest plot of operative time. **(b)** The summary comparison plot for operative time.

For operative time, the evidence network included CC, ES, and MIPS only. The forest plot showed that operative time was significantly shorter with ES and MIPS than with CC (Figure 6a). The summary comparison plot further indicated that, compared with CC, ES reduced operative time (MD = −1.41, 95% CI −2.00 to −0.82), and the reduction was larger for MIPS (MD = −2.46, 95% CI −3.45 to −1.46) (Figure 6b). In addition, operative time was significantly shorter with MIPS than with ES (MD = −1.04, 95% CI −2.04 to −0.05). The consistency test did not suggest significant inconsistency between direct and indirect evidence (χ^2^ (1) = 0.01, *P* = 0.925), indicating good model fit and coherence.

#### 3.3.2 Intraoperative blood loss

**Figure 7:**
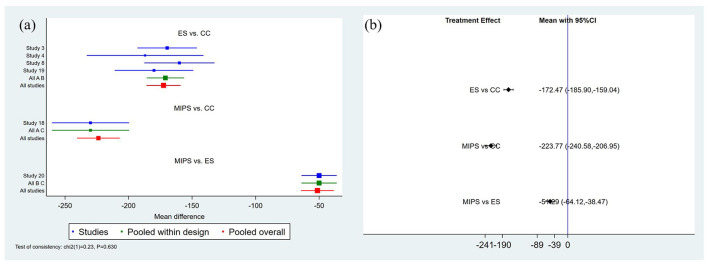
**(a)** The forest plot of intraoperative blood loss. **(b)** The summary comparison plot for intraoperative blood loss.

For intraoperative blood loss, the network again included CC, ES, and MIPS. The forest plot demonstrated that blood loss was significantly lower with ES and MIPS than with CC (Figure 7a). In the summary comparison plot (Figure 7b), ES reduced blood loss compared with CC (MD = −172.47, 95% CI −185.90 to −159.04), and MIPS showed an even larger reduction (MD = −223.77, 95% CI −240.58 to −206.95). Moreover, MIPS was associated with significantly less blood loss than ES (MD = −51.29, 95% CI −64.12 to −38.47). The consistency test indicated no evidence of significant inconsistency (χ^2^ (1) = 0.23, *P* = 0.630), supporting coherence between direct and indirect evidence.

#### 3.3.3 Hematoma clearance rate

**Figure 8:**
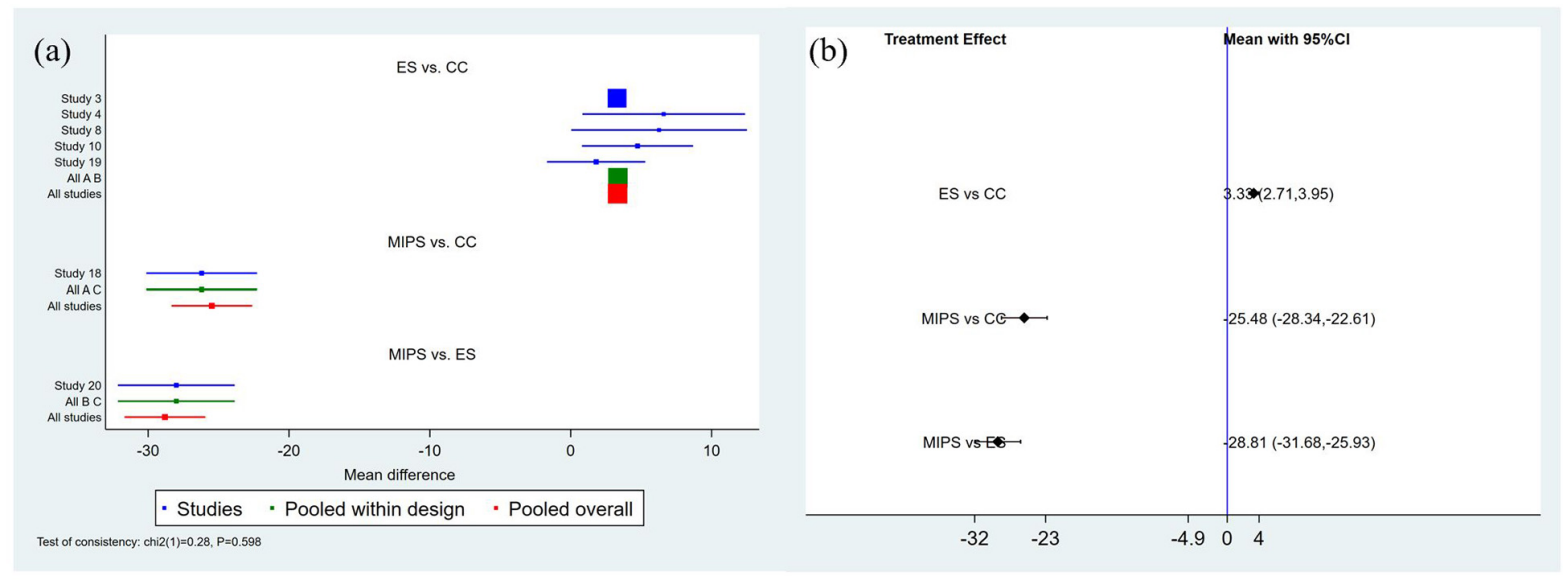
**(a)** The forest plot of hematoma clearance rate. **(b)** The summary comparison plot for hematoma clearance rate.

For hematoma clearance rate, the network included CC, ES, and MIPS. The forest plot suggested that ES achieved a higher clearance rate than CC, whereas MIPS achieved a lower clearance rate than CC (Figure 8a). The summary comparison plot showed that, compared with CC, ES increased hematoma clearance (MD = 3.33, 95% CI 2.71 to 3.95). In contrast, MIPS yielded a lower clearance rate than CC (MD = −25.48, 95% CI −28.34 to −22.61) and was also lower than ES (MD = −28.81, 95% CI −31.68 to −25.93) (Figure 8b). No significant inconsistency was detected (χ^2^ (1) = 0.28, *P* = 0.598), indicating good agreement between direct and indirect evidence.

#### 3.3.4 Length of hospital stay

**Figure 9:**
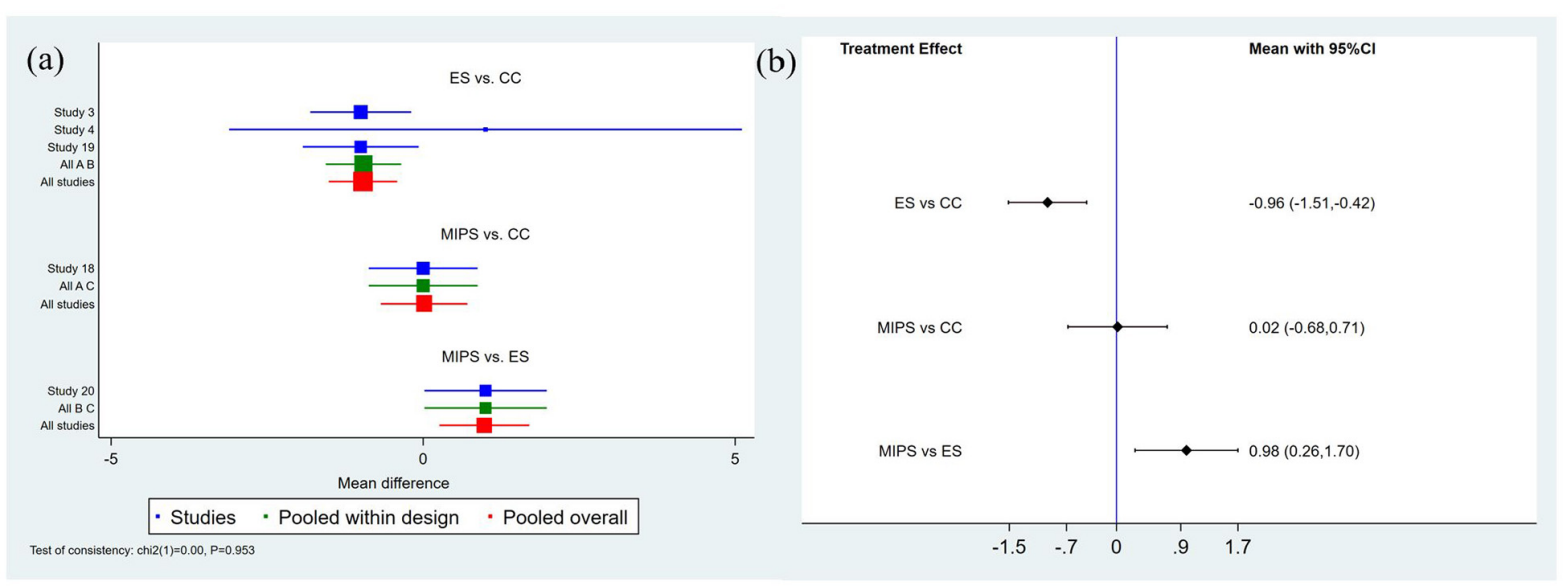
**(a)** The forest plot of length of hospital stay. **(b)** The summary comparison plot for length of hospital stay.

For length of hospital stay, the network included CC, ES, and MIPS. The forest plot indicated that ES was associated with a shorter hospital stay than CC, whereas differences between MIPS and CC were not apparent (Figure 9a). The summary comparison plot showed that, compared with CC, ES significantly shortened hospital stay (MD = −0.96, 95% CI −1.51 to −0.42). In contrast, MIPS did not differ significantly from CC (MD = 0.02, 95% CI −0.68 to 0.71). Additionally, hospital stay was longer with MIPS than with ES (MD = 0.98, 95% CI 0.26 to 1.70) (Figure 9b). The consistency test suggested no evidence of significant inconsistency (χ^2^ (1) = 0.00, *P* = 0.953), supporting coherence of the network estimates.

### 3.4 Sensitivity analyses

Sensitivity analyses were conducted to evaluate the robustness of the main findings. After re-evaluating disease classification, all retained randomized controlled trials were confirmed to involve spontaneous intracerebral hemorrhage rather than subdural hemorrhage. After excluding studies judged to be at high risk of bias, the overall direction of treatment effects for 6-month mortality and good functional outcome remained generally consistent with the primary analysis (Supplementary Figure 4). Functional outcome definitions and major clinical effect modifiers, including hematoma location, baseline severity, hematoma volume, and timing of intervention, were further reviewed qualitatively. Formal subgroup NMA or meta-regression was not feasible because of sparse data and incomplete reporting across treatment comparisons. Therefore, these sensitivity findings should be interpreted as supportive rather than definitive.

### 3.5 Certainty of evidence assessment

A qualitative certainty-of-evidence assessment based on GRADE principles is summarized in Supplementary Table 3. Overall, the certainty of evidence ranged from moderate to very low across outcomes. Evidence certainty for 6-month mortality was judged to be very low because of wide confidence intervals, sparse treatment networks, and uncertainty in indirect comparisons, particularly for decompressive craniectomy (DC). The certainty of evidence for good functional outcome at 6 months was considered low because of clinical heterogeneity, variability in functional outcome definitions, and potential transitivity concerns. For perioperative outcomes, including operative time and intraoperative blood loss, certainty was generally rated as moderate because effect directions were relatively consistent despite limited study numbers and procedural heterogeneity.

## 4 Discussion

In this systematic review and network meta-analysis restricted to randomized controlled trials, we compared conservative medical treatment (CMT) and four surgical strategies (DC, CC, ES, and MIPS) for spontaneous ICH across 6-month outcomes and key perioperative metrics. Overall, our findings suggest potential trade-offs between long-term prognosis (mortality and functional recovery) and perioperative burden (operative time, blood loss, length of stay, and hematoma clearance), providing a structured evidence base to support individualized decision-making.

For the primary outcomes, MIPS and ES ranked highest for good functional outcome at 6 months, and both showed statistically significant advantages over CC and CMT. No significant difference was detected between MIPS and ES, suggesting broadly comparable functional benefits based on the available RCT evidence. These findings are broadly consistent with the STICH and STICH II trials, which reported limited overall benefit of conventional craniotomy compared with conservative management in heterogeneous spontaneous supratentorial ICH populations, although selected subgroups may derive benefit from surgical evacuation (2, 32). In contrast, estimates involving DC were imprecise with wide confidence intervals, indicating that current RCT evidence is insufficient to draw firm conclusions about DC with respect to functional recovery.

For 6-month mortality, DC had the highest SUCRA ranking, with CC and ES ranked next. However, most pairwise comparisons were not statistically significant, reflecting limited precision and uncertainty. The comparison between CC and CMT approached statistical significance, suggesting a possible mortality benefit of CC vs. CMT, but this result should be interpreted cautiously given the borderline confidence interval and the broader uncertainty across the network.

Perioperative outcomes further illuminated clinically relevant trade-offs. Both ES and MIPS were associated with shorter operative time and less intraoperative blood loss than CC, with MIPS showing the largest reductions. These findings are consistent with the minimally invasive nature of ES and MIPS and may be particularly relevant for patients at higher perioperative risk. The favorable perioperative profiles and functional rankings observed for minimally invasive approaches in the present study are also partially consistent with the MISTIE III trial and other contemporary studies suggesting that minimally invasive hematoma evacuation may reduce perihematomal injury and surgical trauma while preserving neurological function in selected patients (19). However, for hematoma clearance, ES achieved a higher clearance rate than CC, whereas MIPS had substantially lower clearance than both CC and ES. This suggests that the perioperative advantages of MIPS may come at the cost of less complete hematoma evacuation, which could influence postoperative course, reintervention needs, and overall recovery in certain patients (38). For length of hospital stay, ES shortened hospitalization compared with CC, while MIPS did not differ from CC and was longer than ES, indicating that shorter procedures and lower blood loss do not necessarily translate into shorter admissions and that postoperative management strategies and residual hematoma burden may play an important role.

Taken together, ES appears to offer a comparatively balanced profile across hematoma clearance, hospital stay, and perioperative burden, whereas MIPS provides the greatest reductions in operative time and blood loss but shows disadvantages in hematoma clearance and does not consistently shorten hospitalization. Although DC demonstrated the highest probabilistic ranking for mortality reduction, this finding should be interpreted cautiously because most pairwise comparisons were not statistically significant and confidence intervals remained wide. Therefore, the SUCRA ranking should not be interpreted as definitive evidence of clinical superiority. Current evidence regarding decompressive craniectomy in spontaneous ICH remains controversial, particularly because potential survival benefits may not necessarily translate into improved long-term functional recovery, and patient selection criteria remain incompletely defined in contemporary literature.

This study has several strengths. First, it addresses a clinically important and high-stakes condition for which comparative evidence remains limited and practice variation is substantial. Second, by restricting inclusion to RCTs and using NMA to integrate direct and indirect evidence, we were able to compare multiple competing strategies (CMT, DC, CC, ES, and MIPS) within a single coherent framework, generating comprehensive relative effect estimates and probabilistic rankings. Third, we evaluated both long-term outcomes (6-month mortality and functional recovery) and perioperative metrics (hematoma clearance, operative time, blood loss, and length of stay), thereby improving clinical interpretability and highlighting potential trade-offs relevant to real-world decision-making. These findings are also broadly aligned with current international guidelines, which continue to recommend individualized decision-making for spontaneous ICH surgery because high-quality comparative evidence remains limited and heterogeneous across patient populations and surgical techniques (3). Finally, model coherence was supported by global inconsistency testing and DIC comparisons, which generally suggested acceptable agreement between consistency and inconsistency models.

Several limitations should be acknowledged. First, although 18 randomized controlled trials were included, several key comparisons—particularly those involving decompressive craniectomy (DC)—were supported by limited evidence with wide confidence intervals, thereby reducing the precision and certainty of conclusions regarding mortality and functional outcomes. Second, despite qualitative assessment of the transitivity assumption through comparison of major clinical and methodological effect modifiers across studies, residual clinical heterogeneity and potential violations of transitivity cannot be completely excluded. Important factors such as baseline neurological severity, hematoma volume and location, timing of intervention, perioperative management, and surgical technical details (e.g., decompression extent, surgical approach, and drainage strategies) were incompletely or heterogeneously reported across trials. These factors may influence treatment effects and potentially bias indirect comparisons.

Third, although 6-month outcomes were pre-specified as the primary endpoints, variations in functional outcome definitions and assessment scales across studies (e.g., mRS, GOS, and study-specific thresholds) may have reduced comparability between trials. In addition, several potentially important sensitivity analyses, including formal subgroup analyses according to hematoma location, baseline severity, and functional outcome definitions, were limited by incomplete reporting and sparse data across treatment comparisons. Consequently, formal subgroup network meta-analysis or meta-regression analyses were not consistently feasible.

Fourth, several secondary outcome networks consisted of relatively sparse three-node structures (CC, ES, and MIPS) with limited direct evidence for certain comparisons, thereby limiting the discriminatory ability and robustness of SUCRA-based rankings. Importantly, the absence of statistically significant inconsistency does not necessarily confirm complete clinical comparability across treatment nodes. Similarly, SUCRA values reflect probabilistic treatment hierarchy rather than definitive evidence of clinical superiority, particularly in networks characterized by sparse data and wide confidence intervals. Therefore, ranking results should be interpreted cautiously alongside effect estimates, confidence intervals, and the overall certainty of evidence.

In addition, the certainty of evidence for several outcomes remained limited because of imprecision, sparse treatment networks, indirect comparisons, and residual clinical heterogeneity across studies. Finally, assessment of publication bias was inherently limited because the number of studies available for several outcomes was relatively small. Accordingly, the findings of this network meta-analysis—particularly those derived predominantly from indirect evidence or sparse treatment nodes—should be interpreted cautiously and considered hypothesis-generating rather than definitive evidence of superiority.

Future trials should prioritize standardized definitions of CMT and perioperative protocols, harmonize surgical technique reporting, and ensure consistent measurement of functional outcomes and perioperative metrics. Adequately powered, multicenter RCTs are particularly needed to clarify the effects of DC on mortality and functional recovery and to identify patient subgroups most likely to benefit from each strategy, ideally incorporating severity stratification and pre-specified subgroup analyses.

## 5 Conclusion

This network meta-analysis of randomized controlled trials suggests that treatment strategies for spontaneous ICH differ in 6-month outcomes and perioperative metrics. For good functional outcome at 6 months, MIPS and ES showed advantages over CC and CMT. For 6-month mortality, DC demonstrated the highest probabilistic ranking; however, the certainty of evidence remained limited because most pairwise comparisons were not statistically significant. Regarding perioperative outcomes, both MIPS and ES reduced operative time and intraoperative blood loss, with larger reductions for MIPS; ES shortened hospital stay and achieved a relatively higher hematoma clearance rate, whereas MIPS showed substantially lower hematoma clearance. Overall, clinical decision-making should balance long-term outcomes against perioperative trade-offs and be individualized according to disease severity and patient-specific risk profiles. High-quality, standardized RCTs are still needed to confirm mortality differences, clarify the true effect of DC, and better define optimal indications for each surgical strategy.

## Data Availability

The original contributions presented in the study are included in the article/Supplementary material, further inquiries can be directed to the corresponding author.
