# Supplementary material for: Surgical strategies for spontaneous intracerebral hemorrhage: a Bayesian network meta-analysis of randomized controlled trials
Source: Front Neurol. 2026 Jun 16;17:1833237. doi: 10.3389/fneur.2026.1833237 (PMC13314473; doi:10.3389/fneur.2026.1833237)

**Appendix.**

**Supplementary data**

**Search Strategy;**

**PUBMED (1589)**

( ("Hematoma, Subdural"[Mesh] OR "subdural hematoma"[Title/Abstract] OR "subdural haematom*"[Title/Abstract] OR "subdural hemorrhage"[Title/Abstract] OR "subdural haemorrhage"[Title/Abstract] OR "subdural bleed*"[Title/Abstract] OR "subdural hematoma*"[Title/Abstract]) AND (spontaneous[Title/Abstract] OR nontraumatic[Title/Abstract] OR "non-traumatic"[Title/Abstract] OR atraumatic[Title/Abstract] OR "a-traumatic"[Title/Abstract] OR idiopathic[Title/Abstract] OR primary[Title/Abstract]) ) AND ( decompressive craniectomy[Title/Abstract] OR craniectomy[Title/Abstract] OR craniotomy[Title/Abstract] OR "open craniotomy"[Title/Abstract] OR endoscop*[Title/Abstract] OR neuroendoscop*[Title/Abstract] OR "minimally invasive"[Title/Abstract] OR puncture[Title/Abstract] OR aspiration[Title/Abstract] OR trephination[Title/Abstract] OR "burr hole"[Title/Abstract] OR "burr-hole"[Title/Abstract] OR drainage[Title/Abstract] OR "catheter drainage"[Title/Abstract] OR surgery[Title/Abstract] OR surgical[Title/Abstract] OR operation[Title/Abstract] OR "conservative treatment"[Title/Abstract] OR "medical management"[Title/Abstract] OR "nonoperative"[Title/Abstract] )

**Web of science (2174)**

1: subdural hematoma (Topic) OR subdural hemorrhag (Topic) OR subdural bleed (Topic)

2: (((((TS=(spontaneous )) OR TS=(nontraumatic )) OR TS=(non-traumatic)) OR TS=(atraumatic )) OR TS=(idiopathic )) OR TS=(primary)

3: (((((((((((((((TS=(decompressive craniectomy)) OR TS=(craniectomy )) OR TS=(craniotomy )) OR TS=(endoscope)) OR TS=(neuroendoscope)) OR TS=(minimally invasive)) OR TS=(puncture )) OR TS=(aspiration )) OR TS=(trephination )) OR TS=(burr hole)) OR TS=(drainage )) OR TS=(catheter drainage)) OR TS=(surgery )) OR TS=(conservative treatment)) OR TS=(medical management)) OR TS=(nonoperative)

4: #2 AND #1 AND #3

**Cochrane Library (105)**

#1 spontaneous

#2 subdural hematoma

#3 subdural hemorrhag

#4 subdural haemorrhag

#5 nontraumatic

#6 non-traumatic

#7 atraumatic

#8 #2 OR #3 OR #4 OR #5 OR #6 OR #7

#9 #1 AND #8

#10 decompressive craniectomy

#11 craniectomy

#12 craniotomy

#13 endoscope

#14 neuroendoscope

#15 minimally invasive

#16 puncture

#17 burr hole

#18 drainage

#19 catheter drainage

#20 conservative treatment

#21 medical management

#22 nonoperative

#23 surgery

#24 #10 OR #11 OR #12 OR #13 OR #14 OR #15 OR #16 #17 OR #18 OR #19 #20 OR #21 OR #22 OR #23

#25 #9 AND #24

|  | The Global inconsistency | ＞0.05 | DIC of Model of consistency | DIC of Model of inconsistency | The difference is  less than 5 |
| --- | --- | --- | --- | --- | --- |
| Good functional outcome at 6 months | 0.2327 | yes | 30.19246 | 33.16545 | yes |
| 6-month mortality | 0.5667 | yes | 29.06554 | 30.67885 | yes |
| Operative time | 0.3631 | yes | 26.49459 | 28.69821 | yes |
| Intraoperative blood loss | 0.5608 | yes | 18.98324 | 19.87895 | yes |
| Hematoma clearance rate | 0.1841 | yes | 17.02346 | 19.91256 | yes |
| Length of hospital stay | 0.4741 | yes | 24.62346 | 27.95743 | yes |

**Supplementary Table 1.** Assessment of model fit. If the difference of DIC value in two modes is within 5, it means that the data is consistent. DIC, deviance information criterion.

| **Study** | **Intervention** | **Hematoma location** | **Baseline severity** | **ICH volume (mL)** | **Timing of intervention** | **Potential transitivity concern** |
| --- | --- | --- | --- | --- | --- | --- |
| Teernstra 2003 | MIPS/CMT | Supratentorial ICH | Glasgow Eye Motor score 2–10 | 59 | <72 h | Low |
| Hattori 2004 | MIPS/CMT | Putaminal hemorrhage | Neurological grade 2–3 | 44 | <24 h | Low |
| Mendelow 2005 | CC/CMT | Supratentorial ICH | GCS 5–15 | 38 | <72 h | Moderate |
| Pantazis 2006 | CC/CMT | Supratentorial ICH | GCS 7–15 | 56 | <8 h | Moderate |
| Kim 2009 | MIPS/CMT | Spontaneous ICH | Mild-to-moderate neurological deficit | 23 | <168 h | Moderate |
| Zhou 2011 | CC/MIPS | Acute supratentorial ICH | GCS ≥5 | NR | NR | Unclear |
| Mendelow 2013 | CC/CMT | Lobar supratentorial ICH | GCS motor score 5–6 | 41 | <72 h | Moderate |
| Zhang 2014 | CC/ES | Basal ganglia hemorrhage | GCS 6–12 | 60 | <24 h | Low |
| Hanley 2016 | MIPS/CMT | Supratentorial ICH | Moderate severity | 46 | <72 h | Low |
| Feng 2016 | CC/ES | Hypertensive cerebral hemorrhage | GCS >5 | NR | NR | Moderate |
| Bhaskar 2017 | CC/CMT | Large supratentorial ICH | GCS 4–12 | 65 | <72 h | Moderate |
| Rasras 2018 | DC/CC | Deep supratentorial ICH | GCS 8–13 | 47 | NR | Moderate |
| Deng 2022 | MIPS/CMT | Intracerebral hemorrhage | NR | 35 | NR | Unclear |
| Noiphithak 2023 | CC/ES | Supratentorial ICH | Moderate neurological impairment | 50 | <12 h | Low |
| Lv 2023 | CC/ES | Basal ganglia hemorrhage | GCS ≥8 | 30 | <24 h | Low |
| Pradilla 2024 | MIPS/CMT | Lobar ICH | Moderate neurological deficit | 55 | <24 h | Moderate |
| Beck 2024 | DC/CMT | Deep supratentorial ICH | Severe neurological impairment | 57 | <72 h | Moderate |
| Xu 2024 | CC/MIPS/ES | Hypertensive supratentorial ICH | GCS 5–14 | 49 | <36 h | Low |

**Supplementary Table 2. Assessment of Potential Effect Modifiers and Transitivity Across Included Studies**

| **Outcome** | **Studies / Participants** | **Main finding** | **Risk of bias** | **Inconsistency / Transitivity** | **Indirectness** | **Imprecision** | **Publication bias** | **Certainty** | **Reason for rating** |
| --- | --- | --- | --- | --- | --- | --- | --- | --- | --- |
| Good functional outcome at 6 months | 18 RCTs / 4,497 patients | MIPS and ES ranked highest; MIPS/ES favored over CC and CMT | Some concerns | Serious concern | Some concern | Serious for DC; moderate overall | Undetected, but limited power | LOW | Downgraded for clinical heterogeneity, variable outcome definitions, and sparse comparisons involving DC. |
| 6-month mortality | 18 RCTs / 4,497 patients | DC had highest probabilistic ranking; most pairwise comparisons non-significant | Some concerns | Serious concern | Some concern | Very serious | Undetected, but limited power | VERY LOW | Downgraded for wide confidence intervals, sparse DC evidence, and uncertainty in indirect comparisons. |
| Operative time | Surgical RCTs / CC, ES, MIPS network | ES and MIPS reduced operative time versus CC; MIPS showed largest reduction | Some concerns | No statistical inconsistency; clinical heterogeneity possible | Low-to-moderate | Moderate | Limited assessment | MODERATE | Effect direction was consistent, but evidence was downgraded for limited studies and clinical variability. |
| Intraoperative blood loss | Surgical RCTs / CC, ES, MIPS network | ES and MIPS reduced blood loss versus CC; MIPS showed largest reduction | Some concerns | No statistical inconsistency; clinical heterogeneity possible | Low-to-moderate | Moderate | Limited assessment | MODERATE | Large and directionally consistent effects, but downgraded for limited evidence base and perioperative heterogeneity. |
| Hematoma clearance rate | Surgical RCTs / CC, ES, MIPS network | ES showed higher clearance; MIPS showed lower clearance than CC/ES | Some concerns | No statistical inconsistency; clinical heterogeneity possible | Moderate | Moderate | Limited assessment | LOW | Downgraded for heterogeneity in surgical techniques, clearance definitions, and limited direct evidence. |
| Length of hospital stay | Surgical RCTs / CC, ES, MIPS network | ES shortened hospital stay; MIPS did not differ from CC | Some concerns | No statistical inconsistency; clinical heterogeneity possible | Moderate | Moderate | Limited assessment | LOW | Downgraded for limited comparisons, heterogeneous discharge criteria, and perioperative management differences. |

# **Supplementary Table 3. Qualitative Certainty-of-Evidence Assessment Based on GRADE Principles**

**Supplementary Figure 1.** The [forest map](javascript:;) of all primary outcomes.

1. The results of [forest map](javascript:;) for good functional outcome at 6 months.


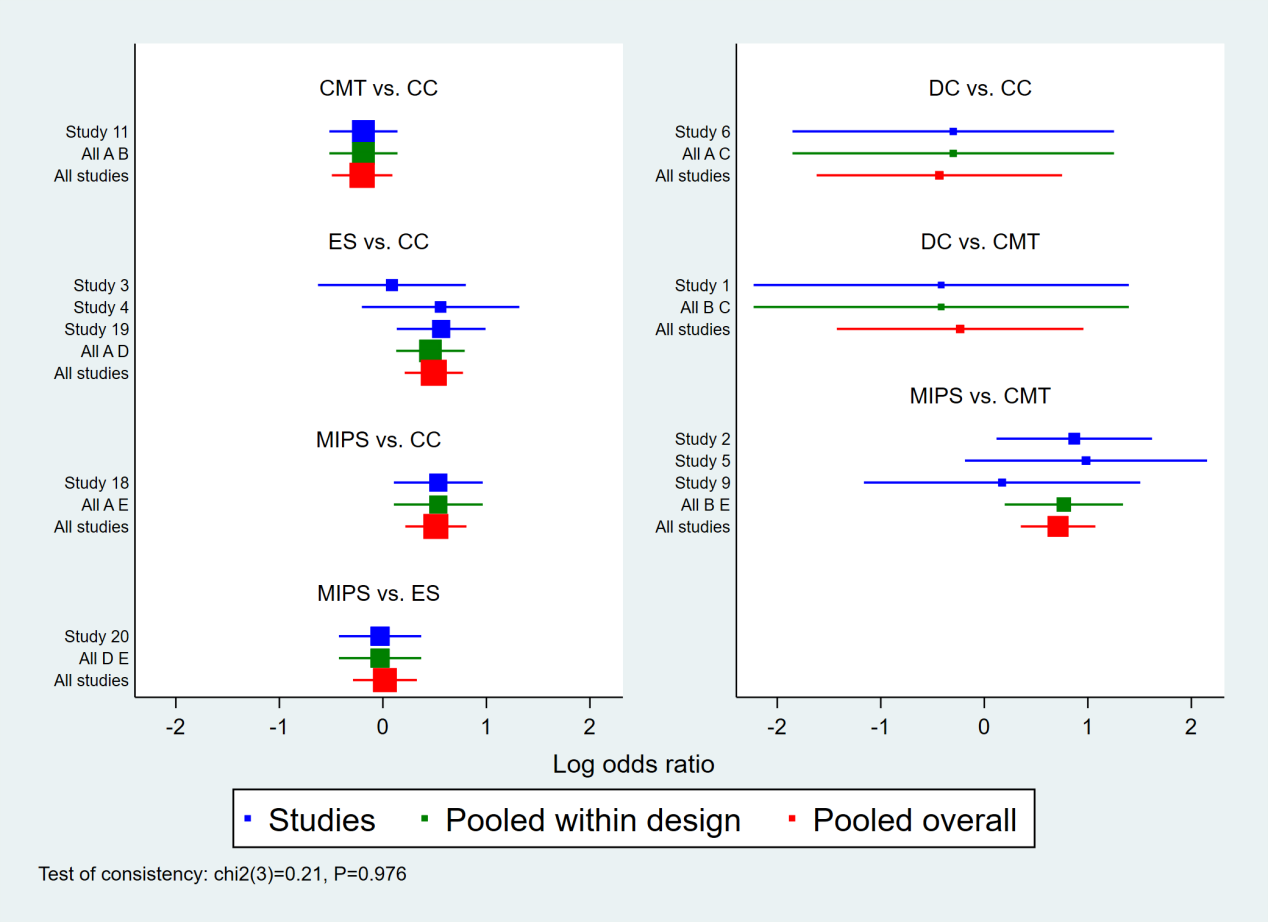


b) The results of [forest map](javascript:;) for 6-month all-cause mortality.


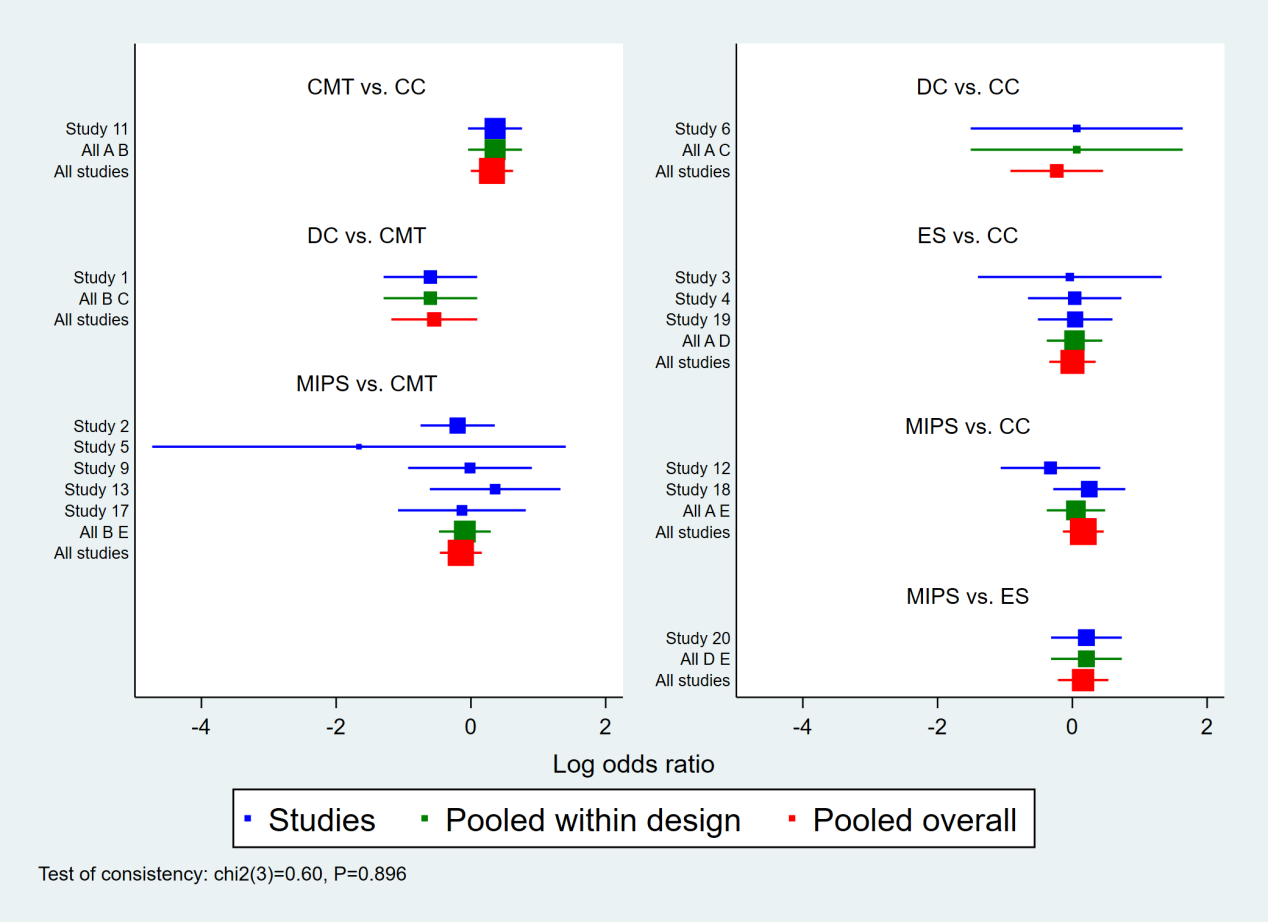


**Supplementary Figure 2.** The network plot of all primary outcomes.

1. Good functional outcome at 6 months


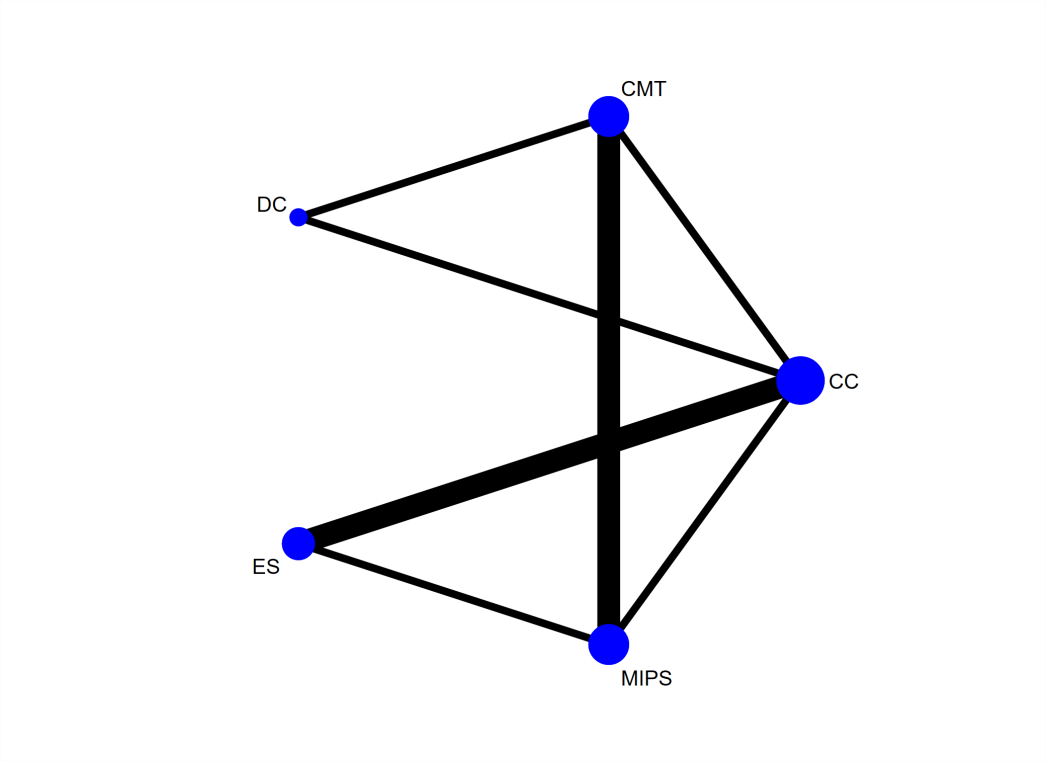


1. 6-month all-cause mortality


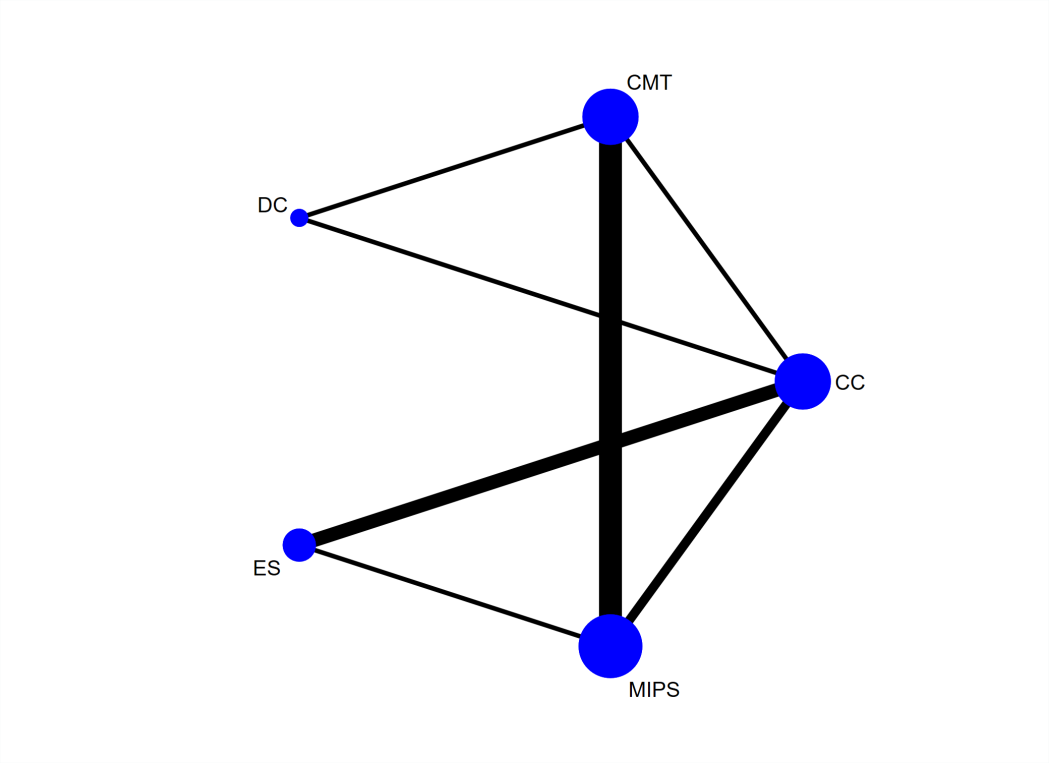


**Supplementary Figure 3.** The Funnel plot of all outcomes.

1. Good functional outcome at 6 months


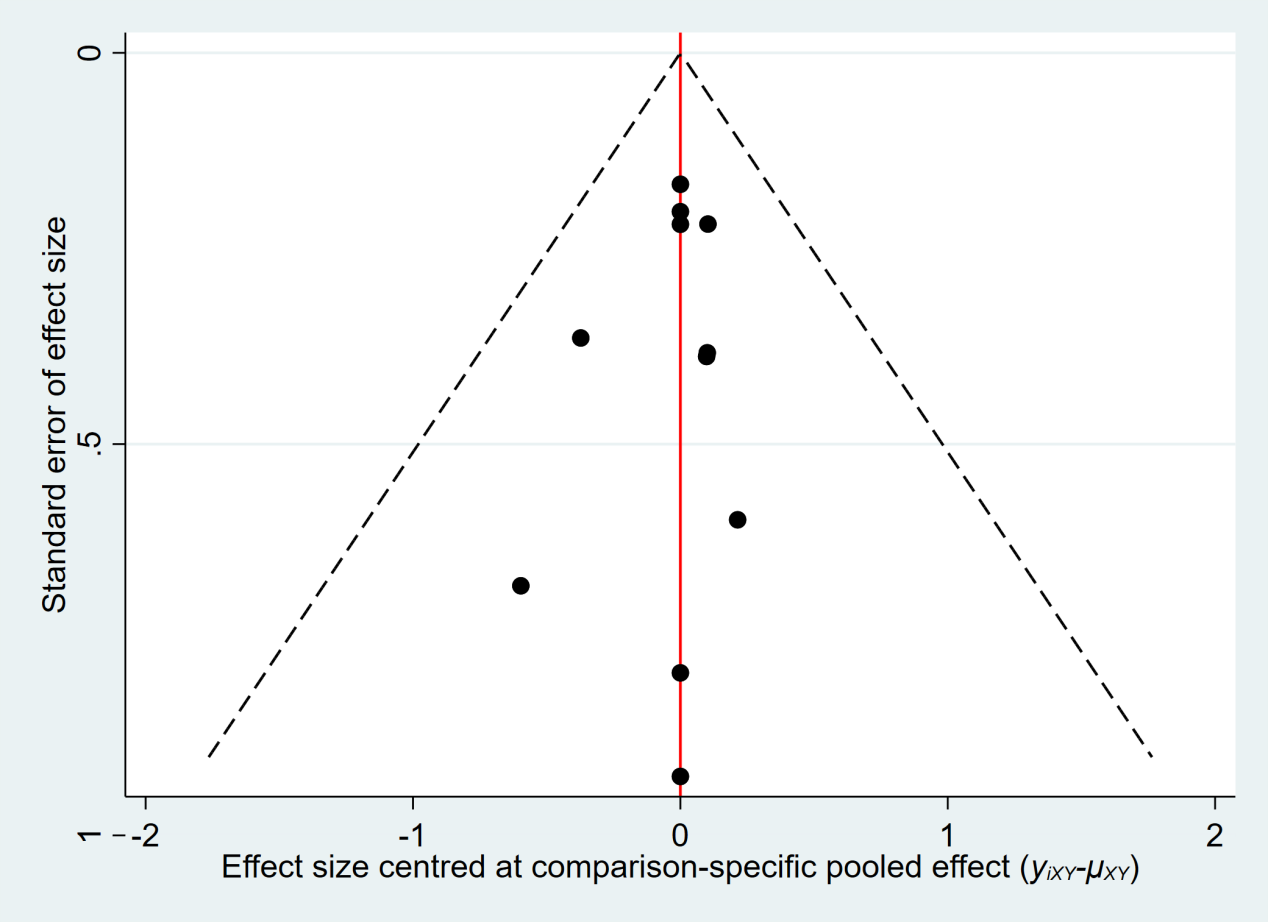


1. 6-month all-cause mortality


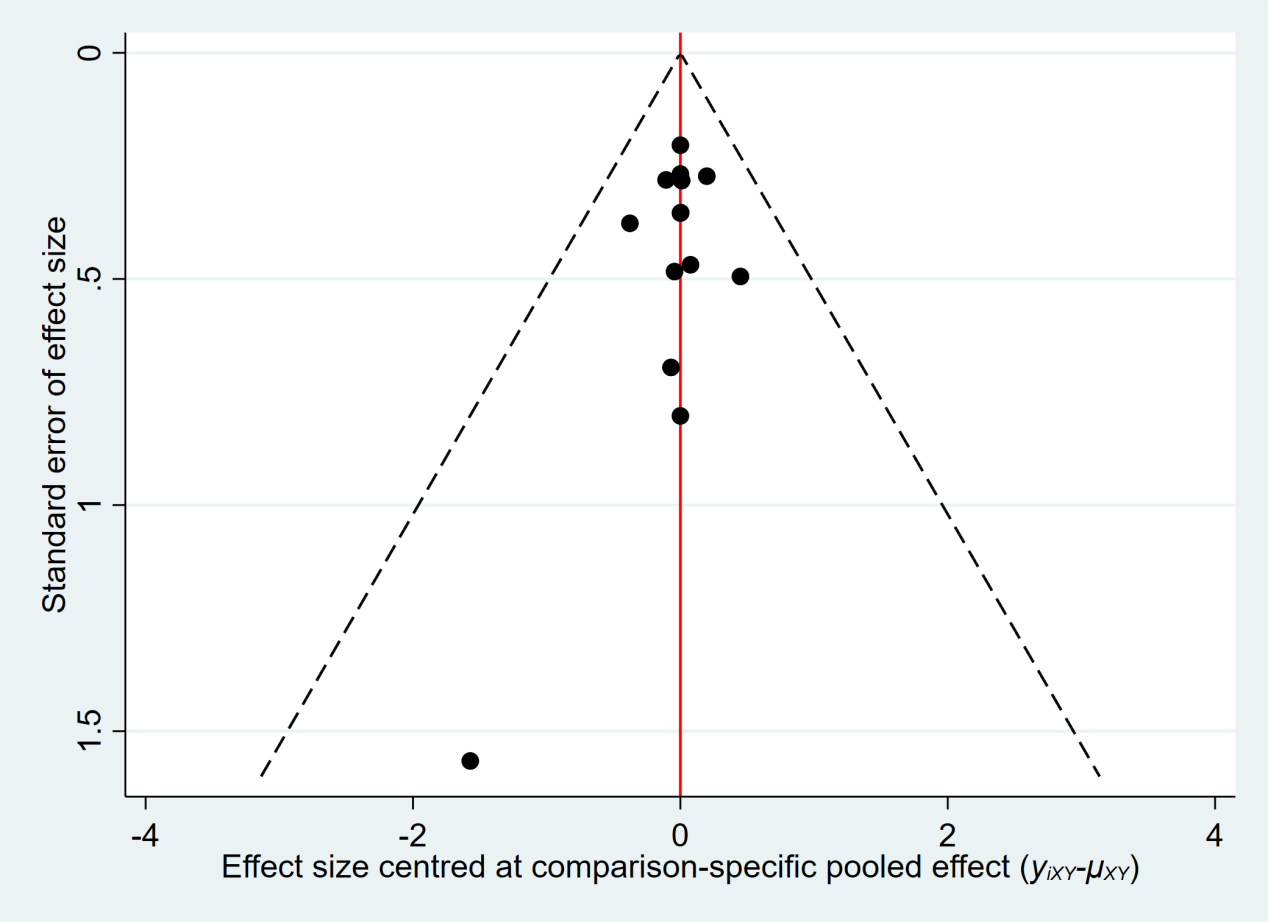


c ) Operative time


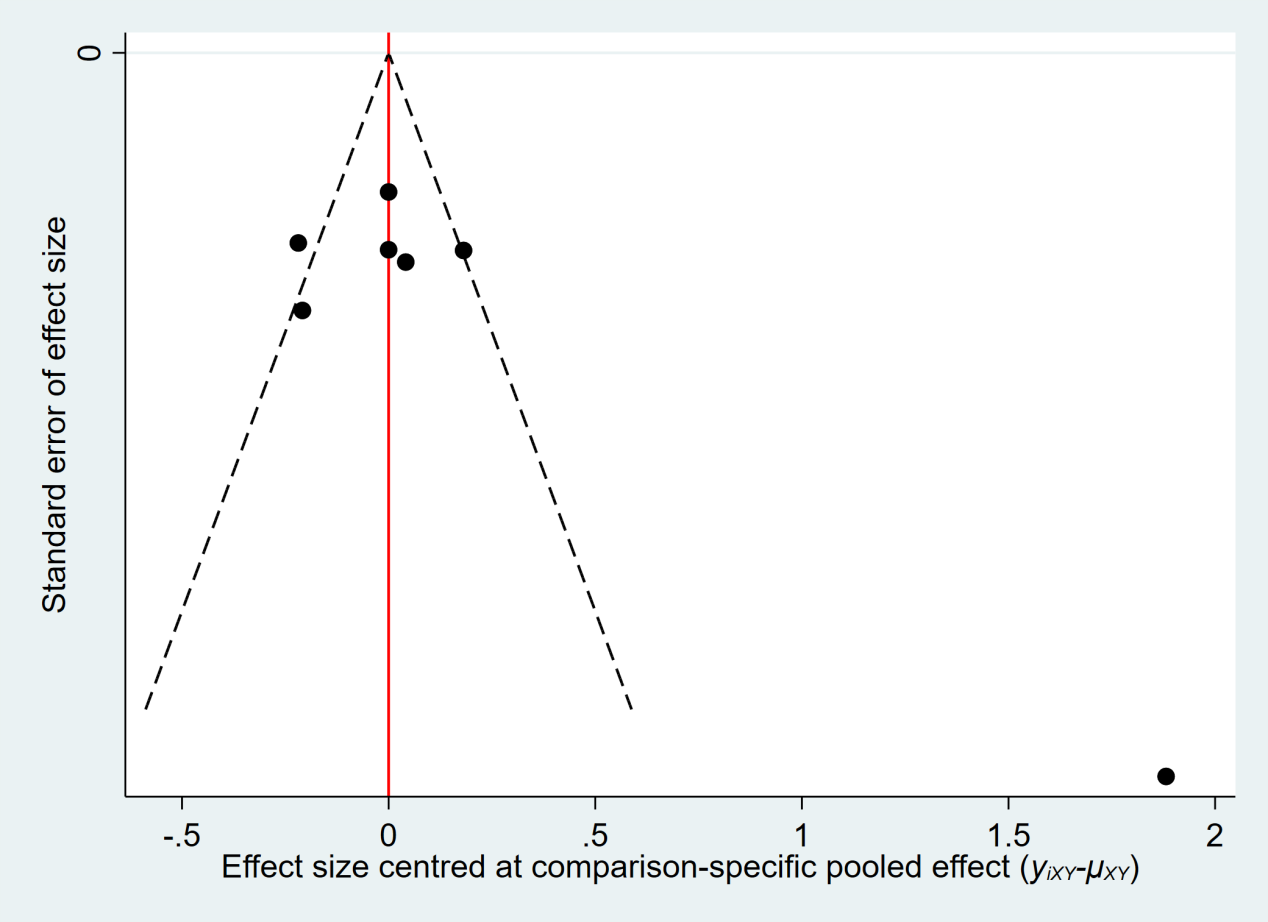


d) Intraoperative blood loss


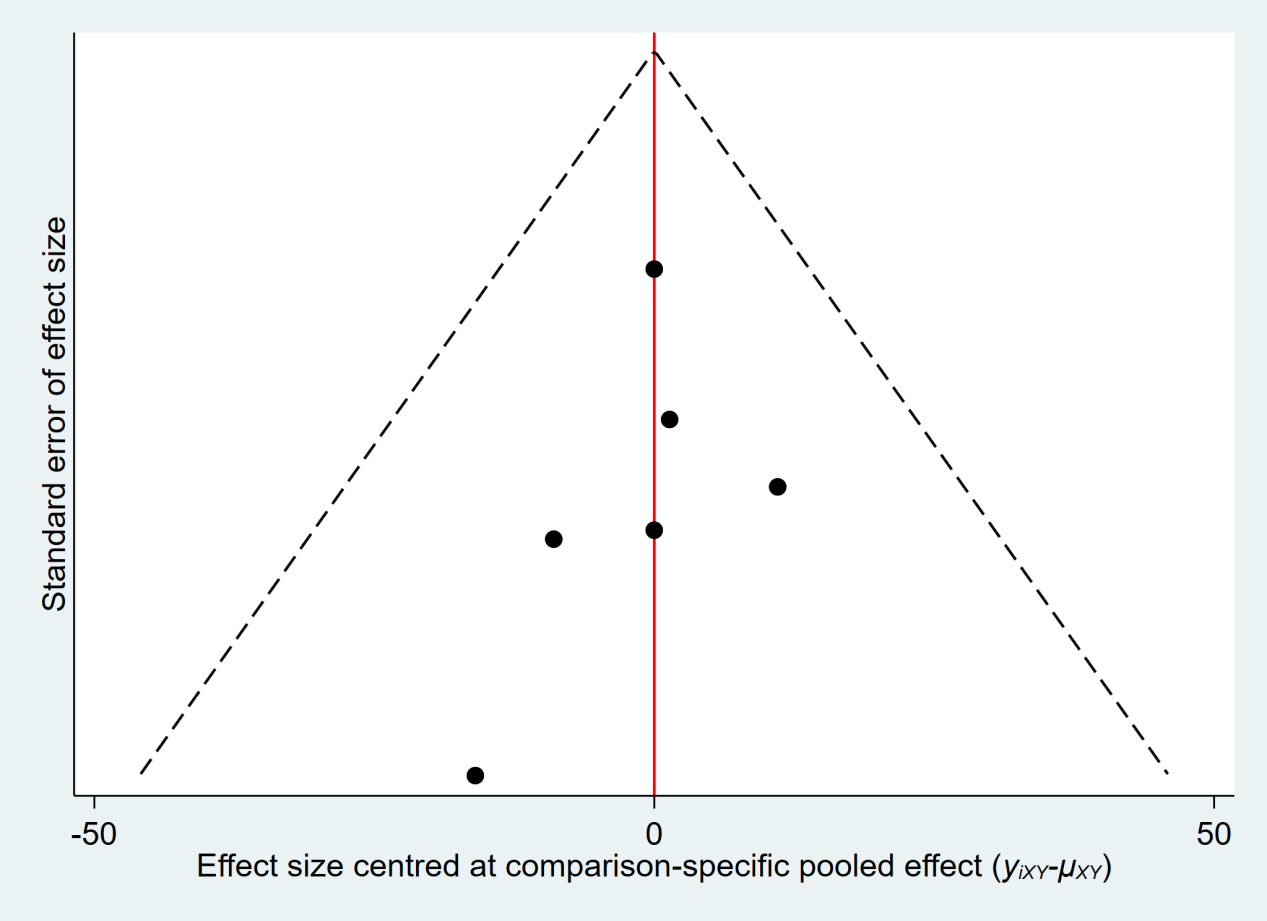


e) Hematoma clearance rate


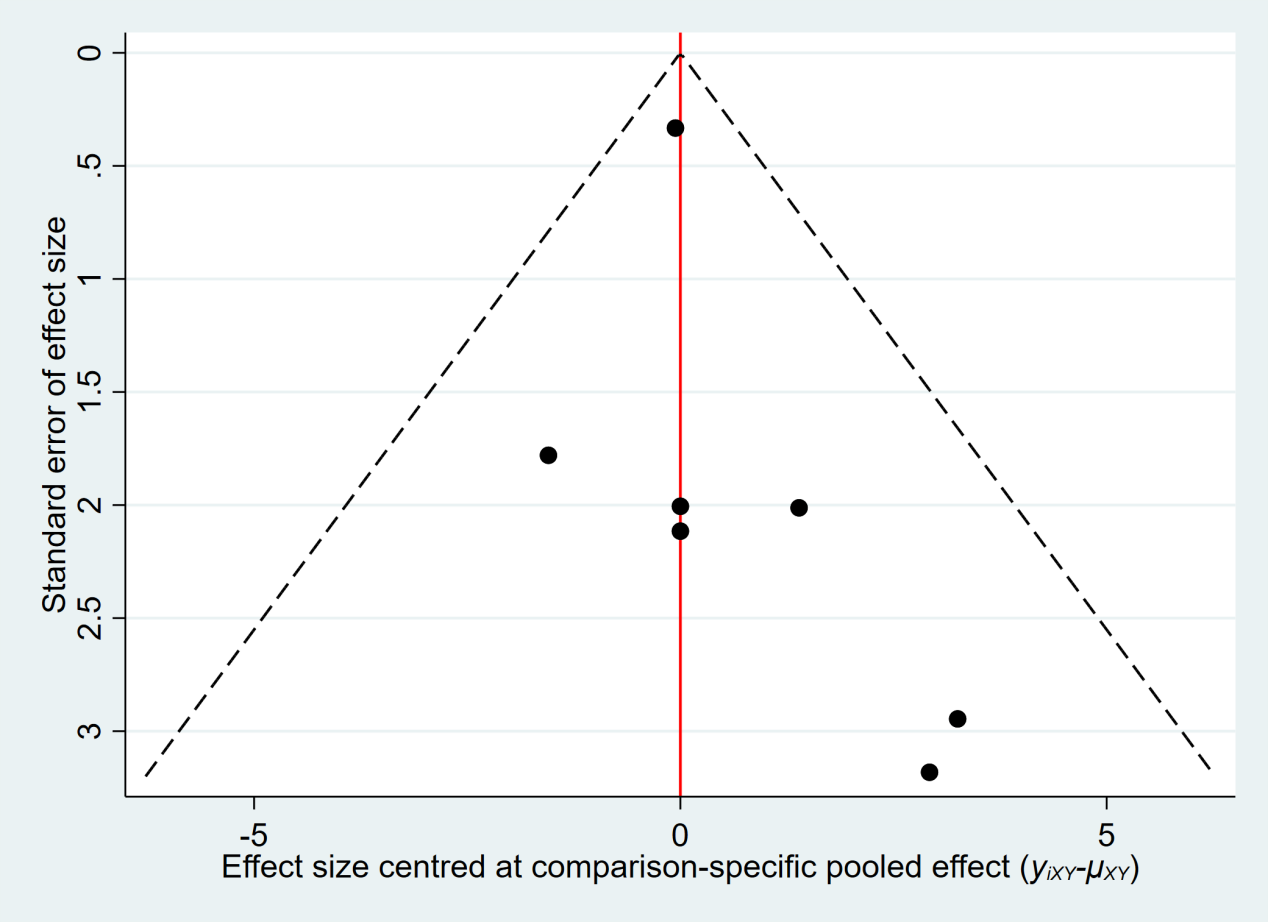


f) Length of hospital stay


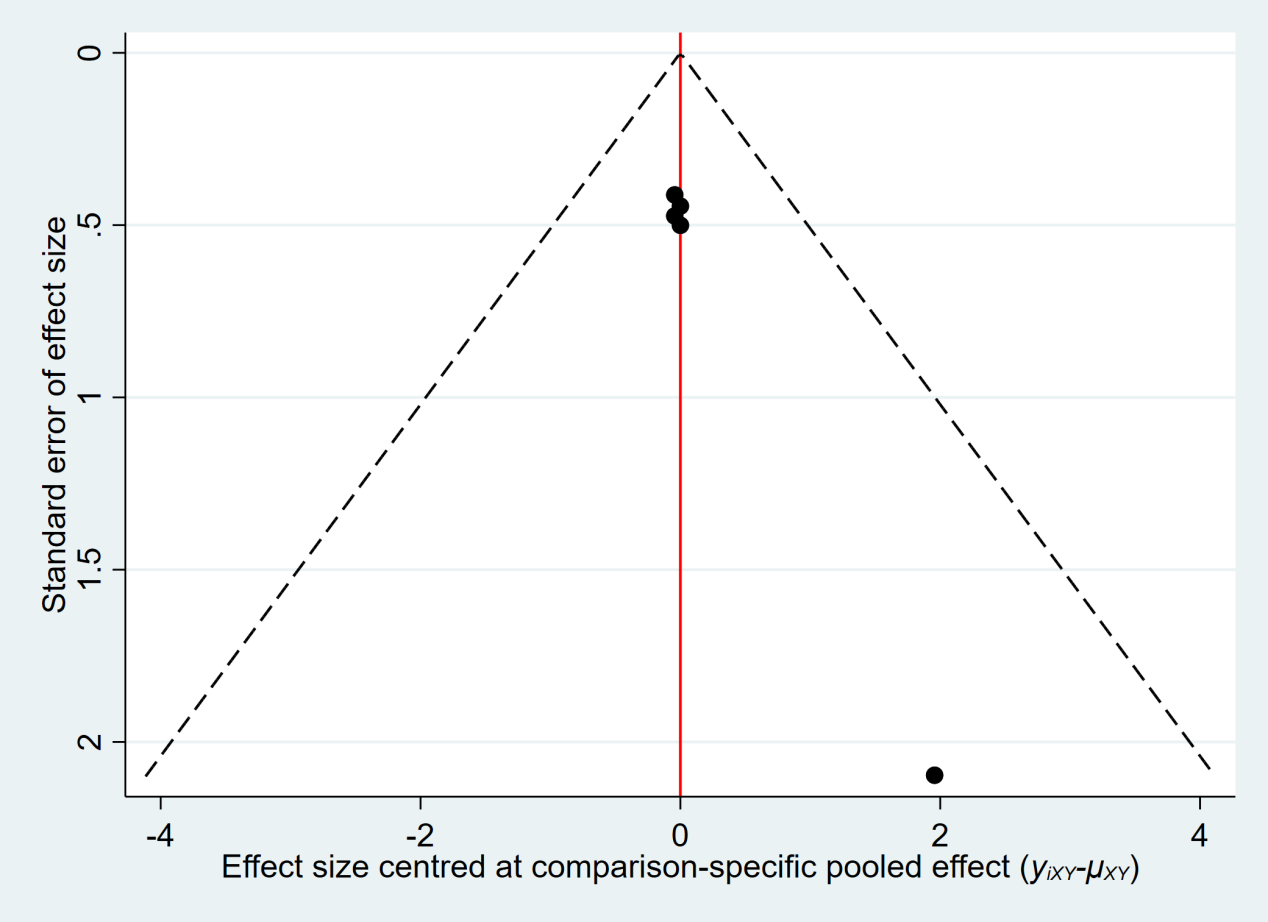


**Supplementary Figure 4.** Sensitivity Analyses for Primary Outcomes After Excluding High-Risk-of-Bias Studies

1. The results of [forest map](javascript:;) for good functional outcome at 6 months.


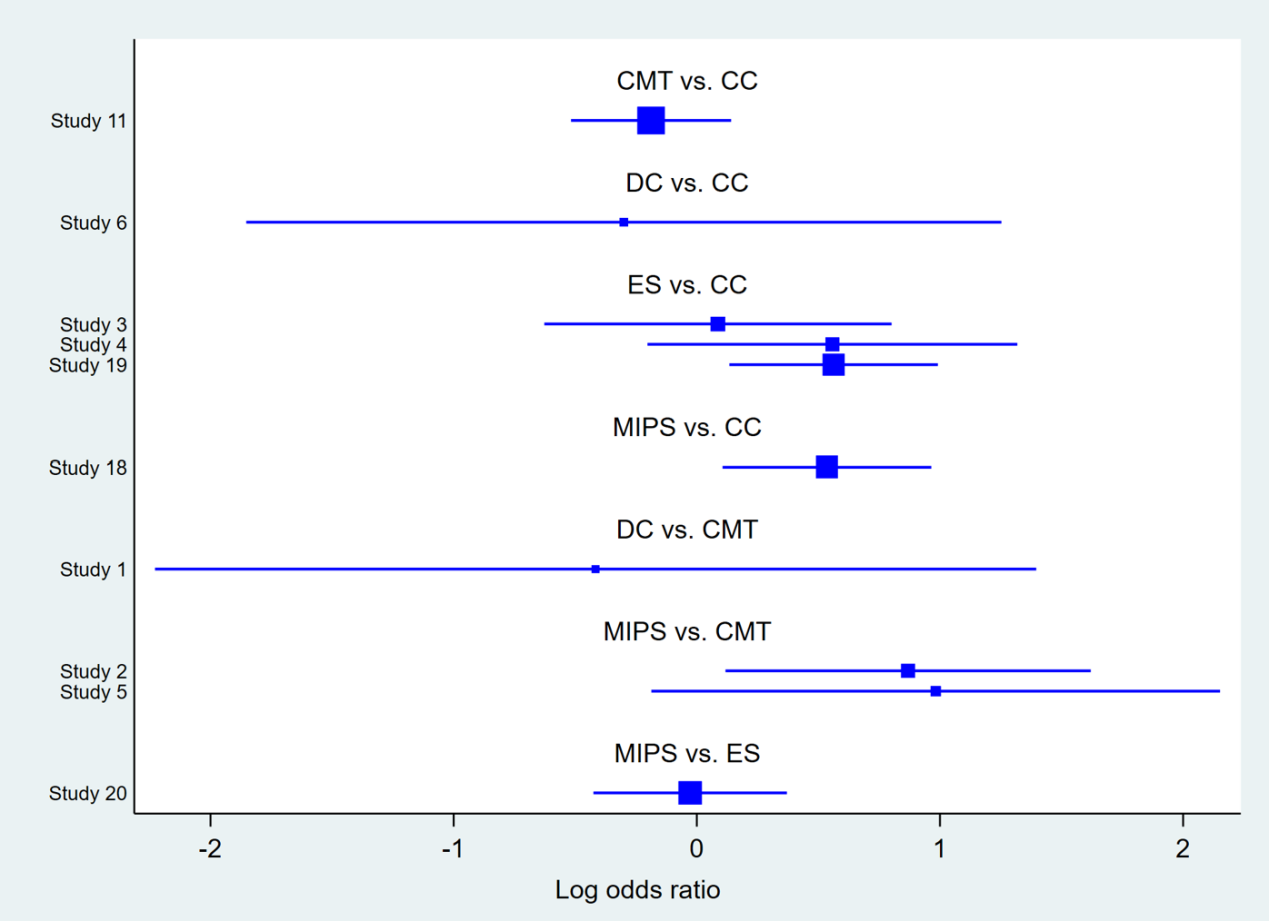


b) The results of [forest map](javascript:;) for 6-month all-cause mortality.


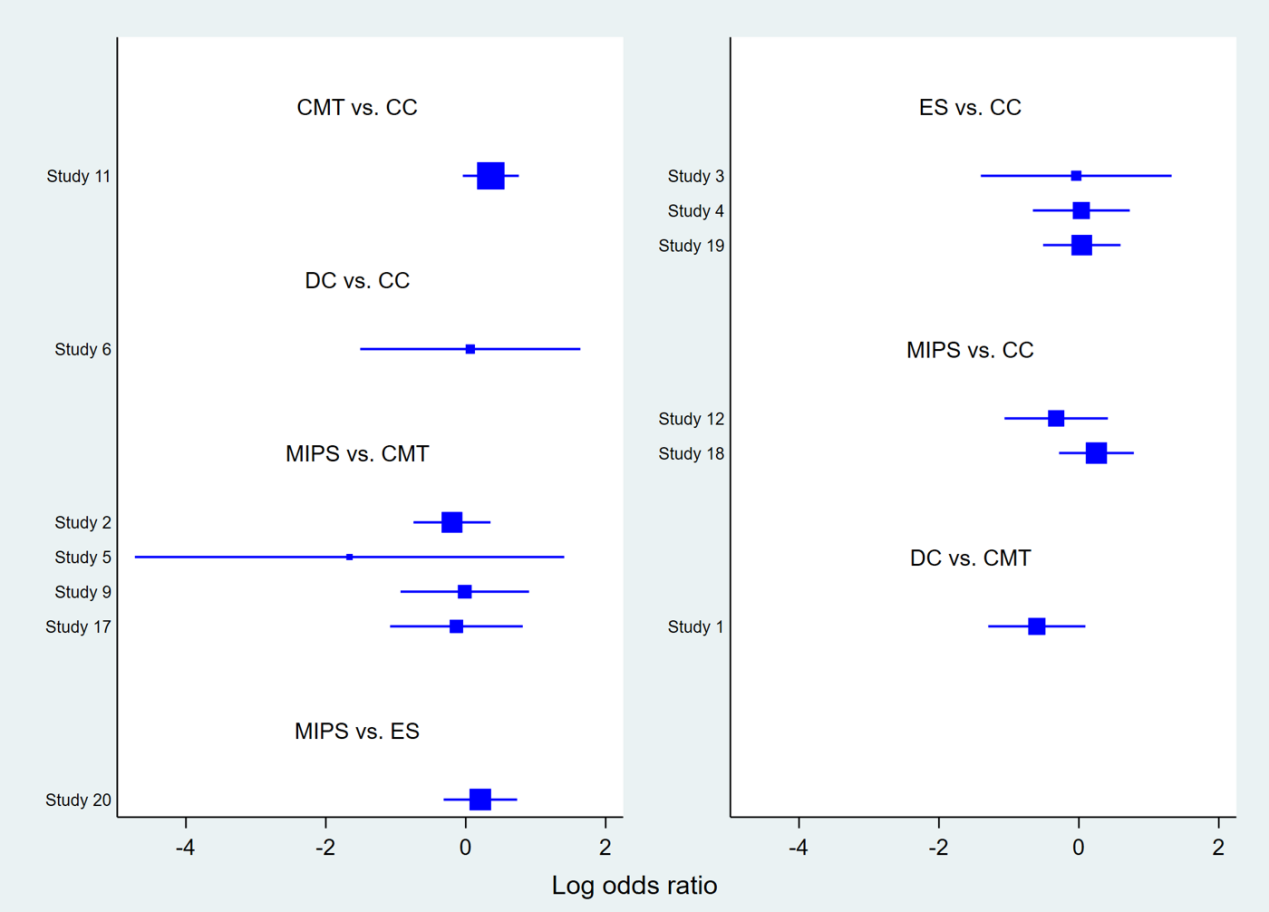

Supplement: Supplementary file 1 [file Supplementary_file_1.docx]
